# Insights into the causal role of diesel exhaust particles in ventricular arrhythmogenesis: protective effects of antioxidant cerium oxide nanoparticles

**DOI:** 10.1186/s12989-025-00649-2

**Published:** 2025-12-25

**Authors:** Freddy G. Ganse, Lena M. Ernst, Cristina Rodríguez, Marisol Ruiz-Meana, Javier Inserte, José Martínez-González, Ana M. Briones, Ana Belén García-Redondo, Marta Consegal, Elisabet Miró-Casas, Laia Yáñez-Bisbe, Aitor Pomposo, Marta Prades-Martínez, Ignacio Ferreira-González, Victor Puntes, Begoña Benito, Antonio Rodríguez-Sinovas

**Affiliations:** 1 Cardiovascular Diseases Research Group, Vall d’Hebron Research Institute (VHIR), Departament de Medicina, Universitat Autònoma de Barcelona, Pg. Vall d’Hebron 119, 08035 Barcelona, Spain; 2 CIBER de Enfermedades Cardiovasculares (CIBERCV), Instituto de Salud Carlos III (ISCIII), Madrid, Spain; 3 Disseny i Farmacodinàmica de Nanopartícules, Vall d’Hebron Research Institute, Barcelona, Spain; 4 Catalan Institute of Nanoscience and Nanotechnology (ICN2), CSIC and BIST, Campus UAB, Bellaterra, 08193 Barcelona, Spain; 5 Institució Catalana de Recerca I Estudis Avançats (ICREA), 08010 Barcelona, Spain; 6 Institut de Recerca Sant Pau (IR SANT PAU), Barcelona, Spain; 7 Instituto de Investigaciones Biomédicas de Barcelona-Consejo Superior de Investigaciones Científicas (IIBB-CSIC), Barcelona, Spain; 8 Department of Pharmacology, Universidad Autónoma de Madrid, Instituto Investigación Hospital Universitario La Paz (IdiPaz), Madrid, Spain; 9 Department of Physiology, Universidad Autónoma de Madrid, Instituto Investigación Hospital Universitario La Paz (IdiPaz), Madrid, Spain; 10 CIBER de Epidemiología y Salud Pública (CIBERESP), Instituto de Salud Carlos III (ISCIII), Madrid, Spain; 11 Networking Research Centre for Bioengineering, Biomaterials and Nanomedicine (CIBER-BBN), Instituto de Salud Carlos III (ISCIII), Madrid, Spain

**Keywords:** Air pollution, Ventricular arrhythmias, Oxidative stress, Fibrosis, Cerium oxide

## Abstract

**Background:**

Epidemiological studies suggest an association between air pollution and ventricular arrhythmias, with reactive oxygen species (ROS) playing a crucial role. However, the causal relationship and long-term effects remain uncertain, and the effectiveness of interventions aimed at reducing ROS requires further investigation. Here we aimed to evaluate the effects of a 3-weeks exposure to diesel exhaust particles (DEPs) on ventricular arrhythmogenesis, explore the underlying mechanisms, and assess the potential of cerium oxide nanoparticles (CeO_2_NP) as a ROS-detoxifying intervention.

**Results:**

Sprague–Dawley rats underwent intratracheal instillation of saline without or with DEPs (7.5 g/Kg for 1–3 weeks). Ventricular arrhythmia inducibility was then assessed in isolated hearts using a protocol of programmed electrical stimulation. Cardiac hypertrophy, collagen content, inflammation and oxidative stress were analyzed using histology, Western blot, RT-qPCR, and measurement of malondialdehyde content. The potential protective effects of CeO_2_NP (0.5 mg/Kg/week, i.p.) were also tested. DEP exposure for 3 weeks increased the incidence and duration of sustained ventricular tachyarrhythmias (VTs), a finding that correlated with a moderate increase in interstitial collagen (from 3.11 ± 0.12% in controls to 4.80 ± 0.21% in DEP-exposed rats, p < 0.001), and an early upregulation in the expression of collagen and other fibrotic and inflammatory markers. These effects associated with prolonged QRS complex and enhanced malondialdehyde content (356.7 ± 21.2 vs. 455.3 ± 17.2 μmol/g tissue, p = 0.0066) after 3 weeks. CeO_2_NP treatment reduced oxidative stress and myocardial fibrosis, reversed electrocardiographic changes and attenuated DEP-induced pro-arrhythmic effects.

**Conclusions:**

DEP exposure increases the incidence and duration of sustained VTs, collagen deposition and oxidative stress in rats. Treatment with CeO_2_NP attenuate these effects, arising as a potential novel strategy to mitigate the deleterious effects of air pollution.

**Supplementary Information:**

The online version contains supplementary material available at 10.1186/s12989-025-00649-2.

## Background

The triad of pollution, climate change, and biodiversity loss constitutes the most pressing global environmental challenge of our time. Among these, pollution raises special concern due to its profound and widespread effects on human health. While recent years have seen reductions in deaths caused by household air pollution and water contamination, these gains have been overshadowed by rising fatalities linked to ambient air pollution and toxic chemical contamination [1]. Deaths attributed to these modern pollution risk factors, unintended consequences of industrialization, have risen by 7% since 2015 and by over 66% since 2000 [1]. Epidemiological evidence further identifies air pollution as one of the leading risk factors for mortality and disability for both males and females worldwide, with significant variations across different geographical regions [2, 3].

Air pollution is composed of a complex mixture of gases (such as ground-level ozone, carbon monoxide, sulfur dioxide, and nitrogen oxides), semi-volatile liquids (methane, benzene, and polyaromatic hydrocarbons) and solid particles (aerosolized soil and dusts and other suspended particles, collectively referred to as particulate matter (PM)) that can be emitted by both natural (forest fires, volcanic eruptions, etc.) and anthropogenic (e.g., industry, traffic, household cooking, etc.) sources [4]. Among these components, PM stands out, given its high toxicity and widespread distribution. PMs are formed by an elemental carbon core surrounded by a variety of chemicals, including sulphates and nitrates, redox-active metals, adsorbed soluble and vaporous hydrocarbons and organic carbon species. Based on their size, PM are categorized into coarse (PM10, 2.5–10 μm), fine (PM2.5, < 2.5 μm), and ultrafine (PM0.1, < 0.1 μm) particles [4].

In Europe, over 790,000 deaths annually can be attributed to air pollution, with about 48% of them being of cardiovascular origin [5]. Indeed, clinical and epidemiological evidence indicates that both short- [6–8] and long-term [8–10] exposure to air pollution, and particularly to PM, is associated with increased cardiovascular mortality. Moreover, PM levels have been linked to a higher incidence of specific cardiovascular disorders, including heart failure [11], myocardial infarction [6] or stroke [12]. In contrast to these relatively well-characterized adverse cardiovascular effects of air pollution, the relationship between air pollution and cardiac arrhythmias remains uncertain [8], with studies yielding conflicting results. Some works have failed to detect significant associations between exposure to air pollution and atrial or ventricular tachyarrhythmias [13, 14], whereas others have reported that both short- [15–17] and long-term [18–20] exposure to PM increases the likelihood of cardiac arrhythmic events.

Experimental studies investigating the effects of air pollution on cardiac arrhythmias in animal models are relatively scarce. Most studies have focused on short-term exposure to diesel exhaust particles (DEPs), rich in PM, in animals with heightened susceptibility. These include models involving coronary artery occlusion [21], isoproterenol-induced heart failure [22] or in hypertensive rats in which arrhythmic events were triggered with aconitate [23]. These effects are associated with prolonged QT intervals and transmembrane action potentials, as well as the activation of CaMKII and the production of reactive oxygen species (ROS) [24, 25].

Given the inconsistencies in clinical and epidemiological studies linking air pollution to cardiac arrhythmias and the limited scope of experimental research, lacking a clear cause-effect relationship and preventive strategies demonstrated thus far, we developed an experimental model of DEP exposure in rats with the following aims: (1) to analyze the effects of intratracheal instillation of DEPs for 3 weeks on ventricular arrhythmogenesis; and (2) to investigate the underlying mechanisms. Additionally, and as previous studies have linked the effects of air pollution to systemic and local inflammation as well as oxidative stress [8, 26, 27], we also investigated whether the effects of DEP exposure observed in our study were associated with this phenomena. In this context, rare-earth cerium oxide nanoparticles (CeO_2_NP) have emerged as safe and potent antioxidant and antiinflammatory agents [28]. Cerium oxide nanoparticles, or nanoceria, exhibit defects in their crystal structure, resulting in the coexistence of both Ce^3+^ and Ce^4+^ ions [28]. This unique property grants CeO_2_NP excellent catalytic activity and ROS-buffering capacity, mimicking the function of enzymes such as superoxide dismutase, catalase and peroxidase [28]. Consequently, our third objective was to assess the impact of CeO_2_NP as a ROS-detoxifying strategy.

## Methods

The data supporting the conclusions of this study are available from the corresponding authors. A complete description of the methods used in this work can be found in the Supplemental material. Animal studies complied with European legislation (Directive 2010/63/EU) on the protection of animals used for scientific purposes, with the Guide for the Care and Use of Laboratory Animals published by the US National Institutes of Health (NIH Publication No. 85–23, revised 1996, updated in 2011), and were approved by the Ethics Committee of Vall d’Hebron Research Institute (protocol number CEEA49.20, CEA/11228/P1/1).

### Preparation of diesel exhaust particles (DEPs)

DEPs (SRM-2975; National Institute of Standards and Technology, Gaithersburg, USA) were suspended in 0.9% sterile saline at a stock concentration of 20 mg/mL, vortexed and sonicated to minimize particle aggregation.

### Preparation of CeO_2_NP

Methods for synthesis, conjugation with rat serum albumin and characterization (Supplemental Fig. S1) of CeO_2_NP can be found in the online Supplemental material. Organ distribution and cerium content was quantified by ICP-MS (Agilent 7900 ICP-MS).

### Experimental design and in vivo exposure to diesel exhaust particles and CeO_2_NP

Seventy-five Sprague–Dawley rats (both sexes, in a 1:1 ratio), aged 6 weeks were used throughout the experiment. Following a 4-week acclimatization period, the rats underwent intratracheal instillations of saline containing or not DEPs (7.5 mg/Kg, 0.375 mL/Kg), for one or 3 weeks, three times a week. The DEP dose and treatment regimen were adapted from Soler-Segovia et al. [29, 30], scaled to the body weight of our animals. The influence of CeO_2_ nanoparticles on the effects of DEP exposure was analyzed in additional rats weekly injected with CeO_2_NP (intraperitoneal; 0.5 mg/Kg the first week, 0.25 mg/Kg the second and third weeks, and 0.5 mg/Kg 1 day before sacrifice), beginning at the time of the first DEP exposure (Supplemental Fig. S2A). The dose of CeO_2_ nanoparticles is in the same order of magnitude of that previously used for intravenous administrations in rats [31]. The pro-arrhythmic effects of DEPs were also assessed in rats with myocardial infarction (i.e., subjected to transient coronary occlusion within the 4 weeks prior to DEP exposure) (Supplemental Fig. S2B). Infarctions were induced as previously described [32], and the DEP exposure protocol was performed as described above.

Successful DEP exposure was confirmed in the lungs of all experimental animals by incubation in benzyl alcohol/benzyl benzoate (BA/BB) or after staining with picrosirius red (Supplemental Fig. S2C).

### Cardiac function by echocardiography

Systolic cardiac function was assessed by transthoracic echocardiography using a Vivid Q portable ultrasound system equipped with a 13 MHz i12L-RS probe (GE Healthcare), as previously described [32]. Echocardiographic images were acquired at the end of the 3-week exposure period, 1 day before euthanasia.

### Isolated, Langendorff-perfused, rat heart preparation

Three weeks after DEP or sham exposure, rats (13 weeks of age, males and females weighing 400–500 g and 250–300 g, respectively) were anesthetized with sodium pentobarbital (1.5 g/Kg, IP) and underwent a bilateral thoracotomy. Whole hearts were quickly excised and retrogradely perfused through the aorta with oxygenated (95% O2: 5% CO2) Krebs solution at 37 °C (composition in mmol/L: NaCl 118, KCl 4.7, MgSO_4_ 1.2, CaCl_2_ 1.8, NaHCO_3_ 25, KH_2_PO_4_ 1.2, glucose 11, pH 7.4) in a constant flow Langendorff system, as previously described [33].

### Electrogram recordings

Electrograms were recorded using stainless steel electrodes (Model 6491 unipolar pediatric temporary pacing lead, Medtronic France, Fourmies, France), as previously described [34]. Changes in the duration of the P wave, PR interval, QRS complex, and Hodges corrected QT interval were analyzed in these recordings.

### Electrophysiological studies

**Fig. 1:**
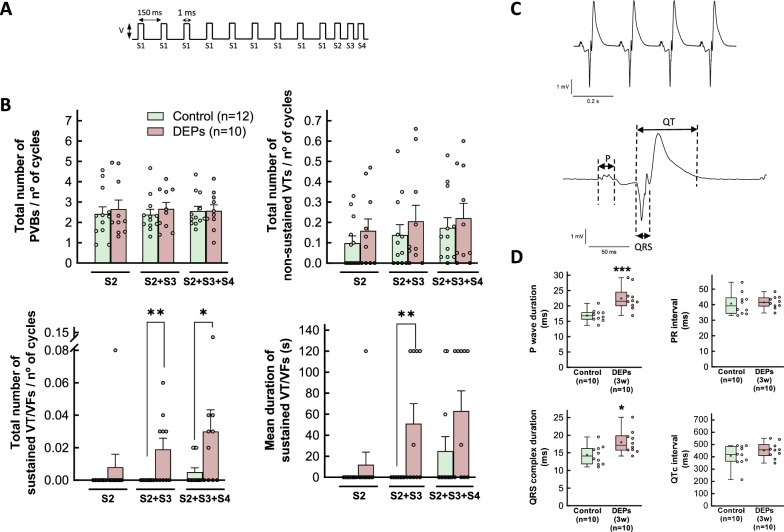
Inducibility of ventricular arrhythmias and electrogram characteristics in hearts from DEP-exposed rats. **A** Outline of the protocol of programmed electrical stimulation used in the study to induce ventricular arrhythmias. A train of 9 stimuli (S1), separated 150 ms, was followed by 1–3 extrastimuli (S2-S4). **B** Number of premature ventricular beats (PVBs), non-sustained tachycardias (VTs) and sustained tachyarrhythmias detected after application of 1, 2 or 3 extrastimuli, expressed relative to the number of stimulation cycles, in isolated rat hearts from animals intratracheally instilled for 3 weeks with saline containing or not DEPs. Additionally, the mean duration of sustained VTs was also determined (lower, right, panel). Significance was tested by Mann–Whitney U test for each number of extrastimuli. *(p < 0.05) and **(p < 0.01) indicate significant differences versus the respective control group. **C** Representative electrogram recording from a heart from a control animal included in the study. The main waves and complexes are shown below. **D** Electrogram characteristics in isolated rat hearts from animals intratracheally instilled for 3 weeks with saline containing or not DEPs. Data are shown as box plot depicting median (horizontal line), mean (+), and individual values (color symbols). Significance tested by Student’s t test. * (p < 0.05) and *** (p < 0.001) indicate significant differences versus control hearts

In all experimental groups, spontaneous ventricular arrhythmias were studied during a 10-min control period. Thereafter, inducible ventricular arrhythmias were triggered using two additional stainless steel electrodes placed at the cardiac apex and a protocol of programmed electrical stimulation, as previously described [34]. This protocol consisted of a train of 9 stimuli (S1), administered at a basic cycle length (BCL) of 150 ms, followed by 1–3 extrastimuli (S2-S4) (Fig. 1a). The number and duration of premature ventricular beats (PVB), non-sustained ventricular tachyarrhythmias (NSVT, defined as those lasting less than 30 s), and sustained ventricular tachyarrhythmias (SVT, lasting more than 30 s) were recorded during the entire protocol. The number of arrhythmic episodes was expressed as the count of events divided by the total stimulation cycles applied to each heart.

### Heart weight to tibia length and interstitial collagen deposition

Cardiac hypertrophy was calculated as the ratio of heart weight to tibia length (HW/TL). Interstitial collagen deposition was determined in cardiac slices stained with picrosirius red [35]. In animals that were submitted to transient coronary occlusion, scar size was determined by quantifying the area of fibrosis stained with picrosirius red, and expressed as a percentage of the total slice area.

### Immunofluorescence analysis of connexin 43 (Cx43) distribution

Cx43 remodeling, including changes in expression and/or distribution, have a major influence on the appearance of cardiac arrhythmias [36]. Accordingly, we assessed Cx43 distribution by confocal laser scan microscopy in cryosections of cardiac samples from control and DEP-treated animals [34]. Additionally, Cx43 expression was analyzed by conventional Western blot as described below.

### Inflammatory cell infiltration

Immunohistochemistry was performed in snap-frozen OCT-embedded cardiac Sects. (4 μm) from additional hearts, using the avidin–biotin method, as described in the online Supplemental material. Sections were incubated with a rabbit antibody raised against CD45 (ab10558, dilution 1:500, Abcam). All staining procedures were performed in triplicate.

### Analysis of myocardial oxidative stress

Oxidative stress was evaluated in myocardial samples from these additional animals by measuring the ratio of reduced to oxidized glutathione (GSH/GSSG) and the concentration of malondialdehyde (MDA), as a marker of lipid peroxidation.

Total GSH concentrations and those of the reduced and oxidized (GSSG) fractions were determined spectrophotometrically at 412 nm in myocardial extracts using a modification of the previously described GSH reductase enzymatic method [37]. A decrease in the GSH/GSSG ratio was used as an indicator of enhanced oxidative stress.

Additionally, lipid peroxidation was assessed by homogenizing myocardial tissue in ice-cold RIPA buffer and measuring MDA levels using a TBARS (TCA Method) Assay Kit (700870, Cayman Chemical), according to manufacturer’s instructions. MDA concentrations were colorimetrically quantified at 430 nm.

### Real time RT-qPCR

Total cardiac RNA was isolated using the TriPure Isolation Reagent (Roche Diagnostics, Indianapolis, USA). DNase I-treated total RNA (1 μg) was reverse-transcribed into cDNA using the High-Capacity cDNA Archive Kit (Applied Biosystems, Foster City, CA) with random hexamers. Quantification of mRNA levels was performed by real-time qPCR using an ABI PRISM 7900HT sequence detection system (Applied Biosystems, Foster City, CA) and specific primers and probes for rat provided by Applied Biosystems (Assay-on-Demand system, see the Supplemental material). Relative mRNA levels were determined using the 2^−ΔΔCt^ method.

### Western blot analysis

Protein expression was analyzed by conventional Western blot, as previously described [35]. Extracts were electrophoretically separated on 10–12% polyacrylamide gels and band intensities were measured by densitometry using the Image Studio Lite software.

### Statistics

Data are expressed as mean ± SEM. Differences in the number of spontaneous or induced ventricular arrhythmias were assessed using nonparametric Mann–Whitney U or Kruskal–Wallis and Dunn’s tests. Additional analyses were performed by two-way ANOVA to determine the effects of previous myocardial infarction and DEP exposure or the impact of sex. Incidences were assessed by χ^2^ or Fisher’s exact tests. Changes in HW/TL, echocardiographic measurements, collagen deposition, inflammatory cell infiltration, RT-qPCR, and Western blot data were analyzed using Student’s t-test or one-way ANOVA with Tukey post-hoc tests. Differences were considered significant when p < 0.05.

## Results

### Arrhythmia inducibility in isolated rat hearts from animals exposed to DEPs

Spontaneous arrhythmias appeared only as occasional isolated PVB, with no differences between experimental groups (data not shown).

Application of the protocol of programmed electrical stimulation to isolated rat hearts from control animals induced only a few arrhythmic events, consisting of PVB along with isolated NSVT. Intratracheal exposure to DEPs did not alter the number of either PVB or NSVT (Fig. 1b). In contrast, DEP exposure significantly increased the incidence (0 out of 12 hearts from control rats vs. 5 out of 10 hearts from DEP-exposed animals after application of 2 extrastimuli, Fisher’s exact test, p = 0.0096), the number and duration of SVT, effect that was particularly evident with two and three extrastimuli (Fig. 1b). These effects were independent of sex (two-way ANOVA, p-NS). On the other hand, DEP exposure did not modify ventricular refractory periods as compared with control animals (Supplemental Fig. S3A).

In animals with a prior myocardial infarction, later DEP exposure did not influence scar size (Supplemental Fig. S3B). Conversely, a previous myocardial infarction further increased arrhythmogenesis in isolated rat hearts, both in the absence and presence of DEP exposure. Indeed, two-way ANOVA revealed a significant effect of myocardial infarction in the number of both NSVT and SVT following application of 1, 2 or 3 extrastimuli (Supplemental Fig. S3C). Additionally, significant effects on arrhythmia duration were observed for both myocardial infarction and DEP exposure after 1 and 2 extrastimuli (Supplemental Fig. S3C). Further analysis using the non-parametric Krustal-Wallis and Dunn’s test showed a significant effect in the number of SVT and in arrhythmia duration in infarcted animals exposed to DEPs (Supplemental Fig. S3C).

### Effects of DEP exposure on cardiac remodeling and the arrhythmogenic substrate

Given that the major proarrhythmic effects of DEPs were observed in healthy animals, subsequent experiments were performed exclusively in rats with no prior infarction. Intratracheal administration of DEPs for 3 weeks did not alter the HW/TL ratio (Supplemental Fig. S4A). These data were confirmed by echocardiographic analyses, which did not show significant changes on cardiac dimensions and function (Supplemental Fig. S4B). Further, no changes were detected in the expression of the hypertrophic markers MYH7 or NNPA between sham animals and those exposed to DEPs (Supplemental Fig. S4C).

**Fig. 2:**
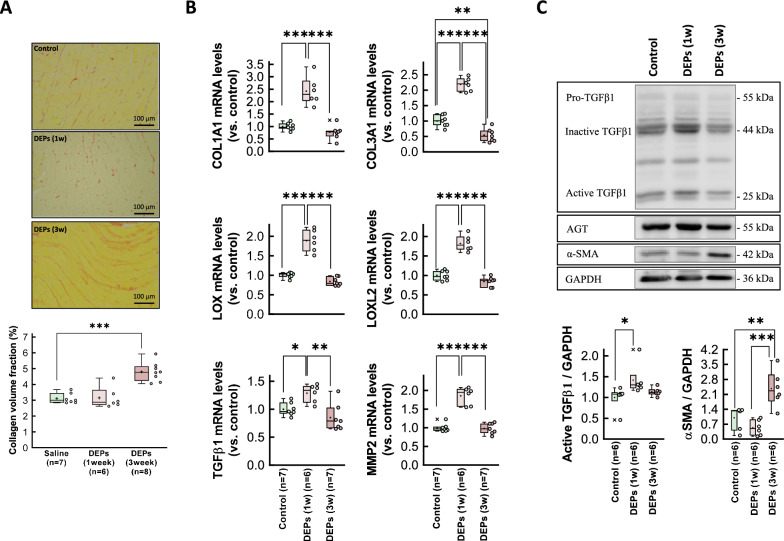
Assessment of fibrosis in hearts from DEP-exposed rats. **A** Interstitial collagen deposition in hearts from rats intratracheally instilled with saline containing or not DEPs after 1 or 3 weeks of exposure. **B** Myocardial levels of mRNAs coding for proteins involved in the fibrotic response (COL1A1, COL3A1, LOX, LOXL2, TGFβ1 and MMP2) analyzed in tissue extracts from the same experimental groups. Values are expressed as fold change with respect to rat adenine phosphoribosyltransferase (APRT), used as a housekeeping gene, and normalized respect to control hearts. **C** Representative Western blot analysis showing expression of TGFβ1, NF-κB, angiotensin receptor (AGT), αSMA and GAPDH, in the same experimental groups. Quantifications of active TGFβ and αSMA are shown below. Data are shown as box plot depicting median (horizontal line), mean (+), and individual values (color symbols). Outliers are marked with “x”, and were also included in the analyses. Significance tested by one-way ANOVA followed by Tukey post-hoc test. * (p < 0.05), ** (p < 0.01) and *** (p < 0.001) indicate significant differences versus indicated groups

In contrast, 3-week exposure to DEPs led to a moderate increase in interstitial collagen deposition (Fig. 2a). This pro-fibrotic response appears to constitute an early response to DEP exposure, as indicated by elevated mRNA levels of the fibrotic markers COL1A1, COL3A1, LOX, LOXL2, and TGFβ1, and of the extracellular matrix-related enzyme MMP2, within 1 week of intratracheal DEP instillation (Fig. 2b). Remarkably, by 3 weeks, expression of these markers returned to control levels (Fig. 2b). Western blot analysis revealed a similar pattern for the active form of TGFβ, while α-smooth muscle actin was enhanced at the end of the exposure period (Fig. 2c).

Western blot analysis of Cx43 demonstrated three specific immunoreactive bands appearing between 41 and 43 kDa, corresponding to different phosphorylation states [36], in myocardial samples from rat hearts. Their quantification did not reveal any difference between control and DEP-exposed animals (Supplemental Fig. S5A). Additionally, confocal analysis did not demonstrate any alteration in Cx43 distribution, with most distributed within the intercalated discs, and presenting only minor lateralization, which was similar in all experimental groups (Supplemental Fig. S5B).

Figure 1c shows a representative cardiac electrogram from an isolated heart included in the control group of animals. Intratracheal instillation of DEPs for 3 weeks resulted in a prolongation of the P wave and the QRS complex (Fig. 1d), along with an increased duration of the QT interval (from 73.23 ± 1.96 to 84.36 ± 2.32 ms in control and DEP-instilled animals, respectively, p = 0.0018). However, when corrected by the Hodges formula, the QTc did not differ between groups (Fig. 1d). No changes were observed in the duration of the PR interval.

### Effects of DEP exposure on cardiac inflammation

**Fig. 3:**
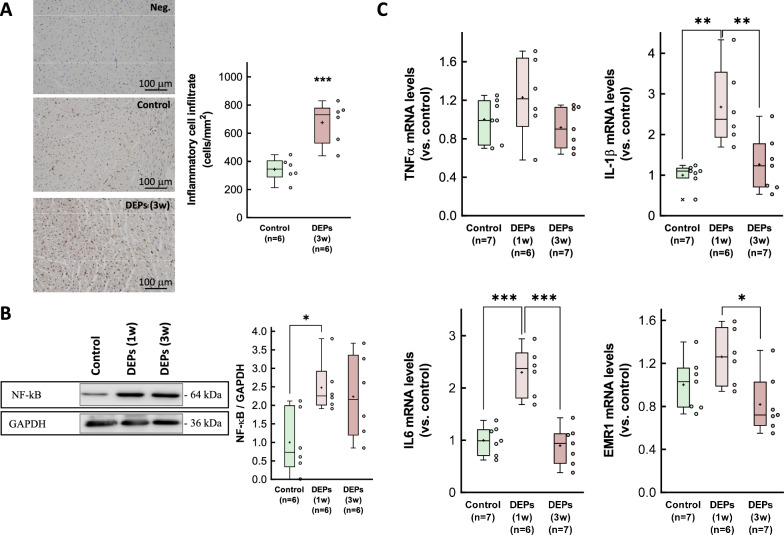
Inflammatory response in hearts from DEP-exposed rats. **A** Representative immunohistochemical images of cardiac slices obtained from a control rat and a DEP-instilled animal, incubated with antibodies raised against CD45, and showing intense immune cell infiltration after 3 weeks of exposure. **B** Expression of NF-κB p65, assessed by Western blot, in myocardial samples from control animals or from rats exposed to DEPs for 1 or 3 weeks. **C** Myocardial levels of mRNAs coding for proteins involved in the inflammatory response (TNFα, IL-1β, IL6 and EMR1) analyzed in tissue extracts from the same experimental groups. Values are expressed as fold change with respect to rat adenine phosphoribosyltransferase (APRT), used as a housekeeping gene, and normalized respect to control hearts. Data are shown as box plot depicting median (horizontal line), mean (+), and individual values (color symbols). Outliers are marked with “x”, and were also included in the analyses. Significance tested by one-way ANOVA followed by Tukey post-hoc test. * (p < 0.05), ** (p < 0.01) and *** (p < 0.001) show significant differences versus indicated groups

Immunohistochemistry using an anti-CD45 antibody revealed an increased inflammatory cell infiltrate in myocardial samples from DEP-exposed rats (Fig. 3a). Further, Western blot analysis demonstrated a significant early increase in NF-κB p65, a transcription factor involved in the development and progression of inflammation in the cardiovascular system [38], following intratracheal DEP instillation, with partial recovery at 3 weeks (Fig. 3b). Consistently with the presence of myocardial inflammation, RT-qPCR analysis showed elevated mRNA levels of IL-1β and IL6 shortly after DEP exposure, with a similar trend for TNFα and the macrophage marker EMR1 (Fig. 3c). Notably, myocardial levels of these inflammatory markers returned to baseline 3 weeks after DEP exposure.

### Myocardial oxidative stress after DEP exposure

**Fig. 4:**
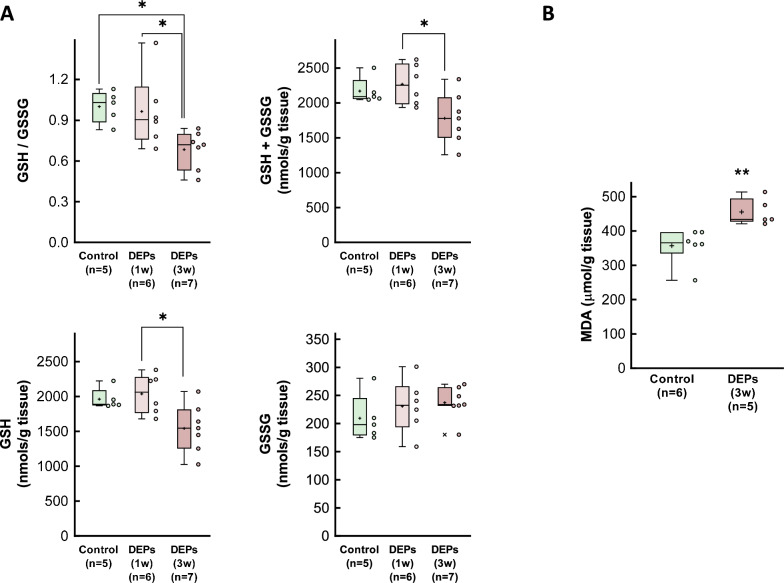
Assessment of oxidative stress in hearts from DEP-exposed rats. **A** Reduced to oxidized glutathione (GSH/GSSG) ratio, together with total GSH concentrations as well as those of the oxidized (GSSG) and reduced (GSH) fractions in myocardial samples from control animals and from rats exposed to DEPs for 1 or 3 weeks. **B** MDA concentrations in myocardial samples from control rats and from animals exposed to DEPs for 3 weeks. Data are shown as box plot depicting median (horizontal line), mean (+), and individual values (color symbols). Outliers are marked with “x”, and were also included in the analyses. Significance tested by one-way ANOVA followed by Tukey post-hoc test. * (p < 0.05) and ** (p < 0.01) show significant differences versus indicated groups

The ratio of reduced to oxidized gluthatione (GSH/GSSG) was significantly diminished in myocardial samples from animals intratracheally exposed to DEPs for 3 weeks, suggesting enhanced oxidative stress (Fig. 4a). Consistently, measurement of myocardial MDA concentrations, a marker of lipid peroxidation, were enhanced at the end of the exposure period (Fig. 4b).

### Activation of cytosolic signaling pathways

Previous studies have linked the effects of PM to the activation of various cytosolic signaling proteins, including ERK1/2, p38 MAPK and Akt [39, 40]. To determine whether the pro-arrhythmic effects of DEP exposure were associated with the activation of these kinases, we assessed their activation and expression by Western blot analysis. Our results showed increased activation (i.e., phosphorylation) of ERK1/2 and decreased phosphorylation of GSK3β 1 week after intratracheal instillation. No changes were observed in other cytosolic signaling molecules, including Akt, p38 MAPK, SMAD2/3 or TAK1 (Supplemental Figs. S6 and S7). In all cases, total protein levels remained unchanged.

### Effects of CeO_2_ nanoparticles on the pro-arrhythmic impact of DEPs

Our findings confirm that exposure to DEPs is associated with increased ROS production and inflammation, contributing to cardiac remodeling, including alterations in ECG characteristics and elevated collagen deposition, ultimately heightening the susceptibility to ventricular arrhythmias in otherwise healthy hearts. Given the pivotal and persistent role of oxidative stress in this process, we subsequently investigated whether treatment with CeO_2_NP, a ROS-detoxifying strategy, could mitigate the effects of DEP exposure on arrhythmia susceptibility.

**Fig. 5:**
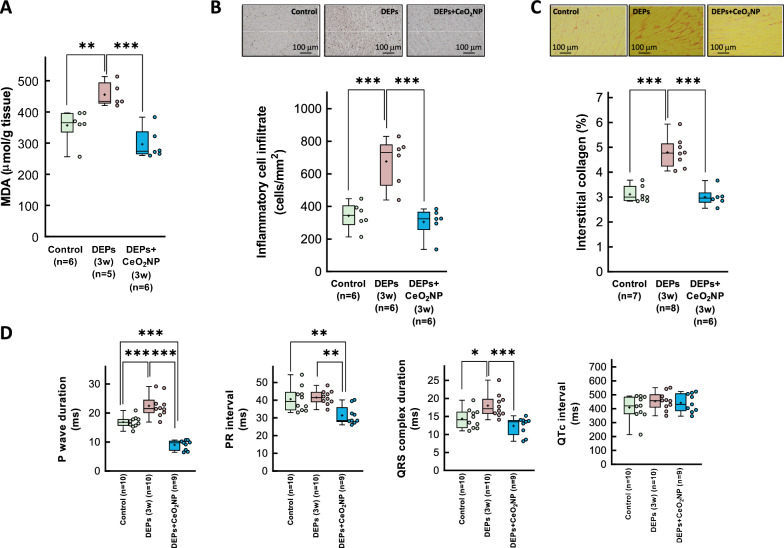
MDA concentrations **A**, immune cell infiltration **B**, interstitial collagen deposition **C** and electrogram characteristics **D**, in isolated rat hearts from animals intratracheally instilled for 3 weeks with saline containing or not DEPs, injected or not with CeO_2_NP. Data are shown as box plot depicting median (horizontal line), mean (+), and individual values (color symbols). Outliers are marked with “x”, and were also included in the analyses. Significance tested by one-way ANOVA followed by Tukey post-hoc test. * (p < 0.05), ** (p < 0.01) and *** (p < 0.001) show significant differences versus indicated groups

**Fig. 6:**
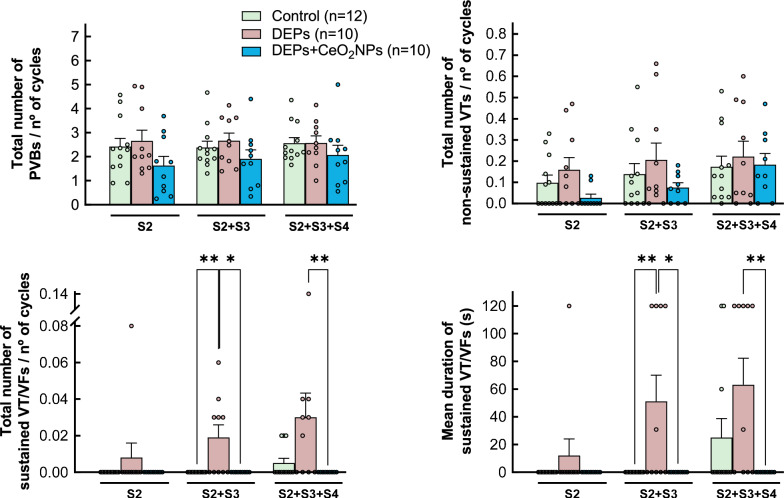
Inducibility of ventricular arrhythmias in hearts from DEP-exposed rats treated with CeO_2_NP. Number of premature ventricular beats (PVBs), non-sustained tachycardias (VTs) and sustained tachyarrhythmias detected after application of 1, 2 or 3 extrastimuli, expressed relative to the number of stimulation cycles, in isolated rat hearts from animals intratracheally instilled for 3 weeks with saline or DEPs, either with or without additional injection of CeO_2_NP. Mean duration of sustained tachyarrhythmias is shown at the right bottom. Significance tested by Kruskal–Wallis and Dunn’s tests. * (p < 0.05) and ** (p < 0.01) show significant differences versus indicated groups

Weekly treatment with a 1 mg/mL CeO₂NP solution (stabilized with 10 mg/mL RSA in 10 mmol/L phosphate buffer, at 0.5 mg/Kg the first week, 0.25 mg/Kg the second and third weeks, and 0.5 mg/Kg 1 day before sacrifice) in rats subjected to DEP exposure restored MDA concentrations (Fig. 5a) and immune cell infiltration (Fig. 5b) to control values. Consequently, CeO₂NPs treatment also normalized interstitial collagen deposition (Fig. 5c) and reversed DEP-induced ECG alterations (Fig. 5d). These effects were accompanied by a trend towards a reduced NSVT incidence and resulted in a complete and statistically significant abolition of SVT compared to DEP-treated rats not receiving CeO₂NPs (0 out of 9 animals, p = 0.0325 vs. DEP-treated rats, Fisher’s exact test) (Fig. 6).

**Fig. 7:**
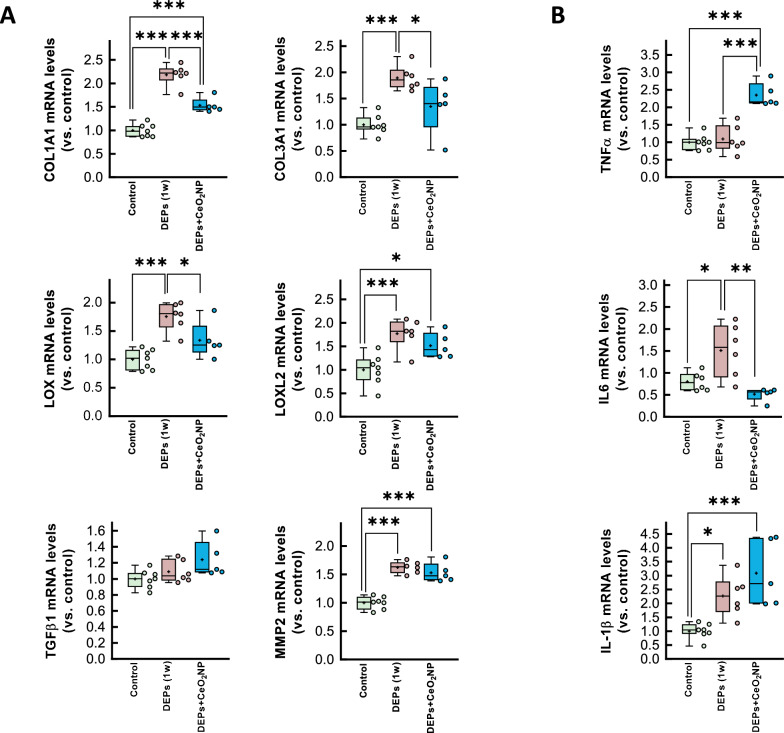
Myocardial levels of mRNAs coding for proteins involved in the fibrotic **A** (COL1A1, COL3A1, LOX, LOXL2, TGFβ1 and MMP2) and inflammatory **B** (TNFα, IL-1β and IL6) responses, analyzed in cardiac extracts from animals intratracheally instilled for 3 weeks with saline containing or not DEPs, injected or not with CeO_2_NP. Data are shown as box plot depicting median (horizontal line), mean (+), and individual values (color symbols). Significance tested by one-way ANOVA followed by Tukey post-hoc test. * (p < 0.05), ** (p < 0.01) and *** (p < 0.001) show significant differences versus indicated groups

Further, RT-qPCR analysis confirmed a reduction in the expression of most fibrotic markers 1 week after DEP exposure (Fig. 7a) along with decreased IL6, though IL-1β mRNA levels remained unchanged (Fig. 7b). Surprisingly, however, CeO₂NPs treatment was associated with enhanced levels of TNFα mRNA levels (Fig. 7b). Remarkably, cerium, detected by ICP-MS, was only present in the liver and the spleen of these animals, with only residual amounts in the heart or other tissues (Supplemental Fig. S8).

## Discussion

This study demonstrates that a 3-week exposure to DEPs induces a pro-arrhythmic effect in isolated rat hearts. This effect was associated with a moderate increase in interstitial collagen deposition, ECG alterations, enhanced myocardial inflammation, sustained increased oxidative stress, and activation of specific cytosolic signaling pathways. Notably, treatment with CeO₂NPs, a well-established ROS scavenger, significantly reduced oxidative stress and inflammation, decreased fibrosis and ECG alterations, and attenuates the pro-arrhythmic effects of DEP exposure. These findings support a causal role for ROS in DEP-induced ventricular arrhythmogenesis.

Evidences linking air pollution to the occurrence of cardiac arrhythmias remains controversial [8]. Clinical and epidemiological studies have yielded contradictory results. In a cohort of patients with implanted cardioverter defibrillators from the Boston metropolitan area, followed for 3.1 years, no statistically significant associations were observed between ventricular arrhythmic episodes and any analyzed pollutant, including PM2.5 on the same or previous days [13]. Similarly, a study in Sweden found no positive association between atrial fibrillation and long-term residential air pollution exposures [14]. In contrast, other studies have demonstrated an increased risk of cardiac arrhythmias following both short- [15–17] and long-term [18–20] exposure to PM.

Most experimental studies have used DEPs as a surrogate for air pollution to assess its impact on cardiac arrhythmias. Previous short-term exposure to DEPs was shown to enhance ventricular arrhythmia duration during ischemia in rats submitted to transient coronary occlusion followed by reperfusion [21]. Furthermore, a single exposure to particulate matter was shown to elevate the risk of aconitine-induced cardiac arrhythmias, including premature ventricular beats and ventricular tachyarrhythmias, in hypertensive rats [23]. However, these studies were conducted under conditions that predispose to arrhythmias. To our knowledge, a single group has investigated the effects of short-term DEP exposure on cardiac arrhythmias in healthy rats, reporting an increased incidence of spontaneous triggered activities and VT 30 min after intratracheal instillation [24, 25]. Our present data demonstrate that DEP exposure not only enhances short-term ventricular arrhythmogenesis but also induces pro-arrhythmic effects after a longer exposure period of 3 weeks in isolated hearts from healthy rats. Specifically, we observed an increase in both the incidence and duration of SVT, the most severe arrhythmic events, which may potentially compromise patient survival. Additionally, prior myocardial infarction further enhanced arrhythmogenesis in isolated rat hearts, effects that tended to be exacerbated by DEP exposure.

While no changes in cardiac dimensions or Cx43 expression and distribution were observed between control and DEP-exposed hearts, the pro-arrhythmic effects detected 3 weeks post-instillation were associated, in our study, with a moderate but significant increase in myocardial fibrosis. Interstitial collagen deposition appears to be an early response, as evidenced by the elevated mRNA levels of fibrosis-associated markers, including COL1A1, COL3A1, and LOX, and of the extracellular matrix-related enzyme MMP2, just 1 week after exposure. Similarly, both mRNA and protein levels of the active form of TGFβ were upregulated soon after DEP exposure. Notably, these transcriptional changes returned to baseline by 3 weeks, suggesting an adaptative response. This pro-fibrotic response observed by us and others [40–42] may contribute to the pro-arrhythmic effects of DEPs. Indeed, previous studies have demonstrated that myocardial fibrosis predisposes individuals to cardiac arrhythmias by altering anisotropic conduction, inducing source-to-sink mismatch, and enhancing refractoriness dispersion [43, 44].

The enhanced fibrotic response may underlie some of the ECG changes we have observed after DEP exposure. Increased P wave duration and dispersion have been correlated with enhanced atrial fibrosis [45], while QRS duration is similarly associated with ventricular fibrosis [46]. In fact, fibrosis can potentially reduce conduction velocity in both cardiac chambers, leading to the prolongation of these two ECG parameters. Our findings align with previous studies demonstrating prolongation of the QRS complex [23, 41] after exposure to DEPs, synthetic residual oil fly ash particles, or particulate matter from ambient air.

Upon inhalation, PM is rapidly phagocytosed by alveolar macrophages, which become over-activated triggering an inflammatory response in the lung [47]. Neutrophil and macrophage activation potentiate cytokine secretion [48] and further recruits additional inflammatory cells, thereby amplifying tissue injury and contributing to systemic and cardiovascular inflammation [47, 49]. Our present findings demonstrate enhanced cardiac inflammation, as demonstrated by an increased inflammatory cell infiltrate in the myocardium. Myocardial inflammation appears to constitute an early response to DEP exposure, paralleling the expression of fibrotic markers. This is supported by elevated NF-κB in Western blot analysis and increased mRNA levels of IL6 and IL-1β 1 week after DEP exposure, with similar trends for TNFα and EMR1. However, similar to fibrosis, an adaptative response seems to occur by 3 weeks, as these mRNA levels return to baseline.

Inflammation goes hand-in-hand with oxidative stress, with inflammatory cells producing ROS, while ROS, in turn, enhance inflammation through NLRP3 inflammasome activation [50], creating a positive feedback that exacerbates tissue injury [49]. Our current findings demonstrate a decreased GSH/GSSG ratio 3 weeks after exposure, accompanied by increased myocardial MDA concentrations, both of which are indicative of heightened oxidative stress and lipid peroxidation. Notably, increased oxidative stress may have significant consequences in sarcolemmal currents that may favor the appearance of arrhythmias [51]. Further, ROS have been shown to promote fibroblast differentiation into myofibroblasts [52], contributing to interstitial fibrosis, as suggested in our study by elevated αSMA levels. In this regard, we observed enhanced ERK1/2 activation in myocardial samples from DEP-exposed animals, supporting previous studies implicating the AngII/ERK1/2/TGFβ1 signaling pathway in PM2.5-induced fibrosis [40]. Furthermore, our Western blot analysis revealed decreased phosphorylation (i.e., enhanced activation) of GSK3β 1 week after intratracheal instillation. Given GSK3β ‘s critical role in mitochondrial regulation [53], this finding aligns with previous reports of mitochondrial dysfunction following DEP exposure [54].

Notably, our findings reveal that the pro-arrhythmic effects of DEPs were reversed by treatment with CeO₂NPs. Indeed, CeO₂NPs have been shown to confer cardioprotective effects against several ROS-dependent pathologies. They attenuated QT prolongation, cardiac enzyme release, oxidative stress and apoptosis in a rat model of doxorubicin-induced cardiomyopathy [55]. Similarly, CeO₂NPs reduced left ventricular dysfunction and dilatation in mice with cardiac-specific expression of monocyte chemoattractant protein (MCP)-1 expression [56], and mitigated cardiomyopathy secondary to obesity induced by a high-fat, high sucrose, diet [57]. Additionally, CeO₂NPs diminished monocrotaline-induced pulmonary hypertension and associated right ventricular hypertrophy in rats [58]. Finally, CeO₂NPs-decorated polycaprolactone (PCL)-gelatin blend nanofibers significantly reduced ROS levels and attenuated agonist-induced hypertrophy in neonatal rat primary cardiomyocytes [59]. To our knowledge, this is the first study to analyze the effects of CeO₂NPs on ventricular arrhythmogenesis.

The antiarrhythmic effects of CeO₂NPs were associated with a reduction in oxidative stress, as indicated by the recovery in MDA levels. This finding underscores not only the potential of CeO₂NPs as a therapeutic strategy but also the critical role of ROS in the harmful cardiovascular effects of air pollution, particularly DEPs. This aligns with previous studies suggesting that antioxidants such as N-acetylcysteine (NAC) may mitigate the adverse cardiac effects of PM [24, 60]. However, the use of NAC as a safe antioxidant has come under debate due to a lack of evidence regarding its antioxidant effects and its poor pharmacology [61]. As expected, the reduction in oxidative stress was accompanied by decreased collagen deposition 3 weeks after exposure, downregulation of fibrosis-related mRNA markers, a decrease in infiltrating inflammatory cells, shortened P wave and QRS durations, and attenuated IL6 expression. Contrarily, however, CeO₂NPs-treated hearts showed an increase in TNFα mRNA levels. The underlying mechanism for this discrepancy remains unclear. In this regard, it is important to note that the biocatalytic ROS-scavenging activity of CeO₂NPs originates from oxygen vacancies (Oᵥ) on their surface, which react directly with reactive oxygen species [62]. The CeO₂ crystal lattice serves as a structural carrier that stabilizes these active sites. However, as with any carrier system, there can be trade-offs. When CeO₂NPs aggregate, the oxygen vacancies become buried within the particle cluster, reducing their accessibility. In such cases, the enlarged aggregates can promote pro-inflammatory responses. In contrast, small, well-dispersed nanoparticles with a high surface density of exposed oxygen vacancies, such as those used in our study, tend to exhibit anti-inflammatory effects [63]. Interestingly, we also previously observed that in healthy control animals, CeO₂NP administration induced mild and transient pro-inflammatory responses [63, 64]. However, under conditions of oxidative stress, the same nanoparticles displayed strong anti-inflammatory properties [62, 63]. Further studies will be conducted to better understand the balance between these pro- and anti-inflammatory effects and the mechanisms underlying this context-dependent response.

As previously reported, intravenously administered cerium oxide nanoparticles (CeO₂NPs) are primarily distributed to the liver and spleen [65, 66], where they are well tolerated [28]. Consistent with these findings, our study also observed a predominant accumulation of CeO₂NPs in these organs, with only a small fraction detected in the lungs 1 day after the final administration. CeO₂NPs are known to gradually build up in the liver and spleen and are slowly eliminated through the hepatobiliary route [65, 67]. Therefore, it is possible that higher concentrations could be present in the lungs at earlier time points following administration. The ROS-scavenging and anti-inflammatory effects of CeO₂NPs are intrinsically catalytic. This means that CeO₂NPs facilitate the recombination of free radicals without being consumed during the reaction. As a result, even low doses can provide long-lasting antioxidant protection [62]. Diesel exhaust particles (DEPs), on the other hand, are well known to trigger lung inflammation, which can become systemic and contribute to cardiovascular damage. In addition, air pollution is known not only to affect the lungs and the heart but also to cause significant harm to the liver [68, 69]. Because the liver is highly sensitive to oxidative stress, it plays a central role in amplifying systemic inflammation [64, 70]. One proposed mechanism suggests that lung injury caused by particulate matter leads to the release of cellular contents and pro-inflammatory mediators, which then trigger liver inflammation and promote systemic inflammatory responses [71, 72]. Kido et al. [73] demonstrated that acute pulmonary inflammation can “spill over” into the bloodstream, leading to systemic inflammation. Cytokines such as interleukin-1β (IL-1β) and interleukin-6 (IL-6) further stimulate hepatic production of acute-phase proteins such as C-reactive protein (CRP), contributing to endothelial dysfunction in systemic blood vessels [73]. Conversely, removal of inflammatory mediators such as IL-6 has been shown to restore vascular endothelial integrity [73]. In this way, CeO₂NPs located in the liver and spleen could still exert protective effects remotely, potentially by limiting systemic inflammation and reducing ROS production and propagation. Although in our study we did not measure circulating cytokines or systemic markers of oxidative stress in rat blood, this represents an important avenue for future research. Characterizing cytokine release kinetics could help clarify the potential systemic actions of CeO₂NPs. Similar remote actions have been described in other models, as in rats with sepsis-induced cerebral injury [74] or in mice with oxalate-induced kidney injury [75]. An additional possibility is that small amounts of CeO_2_NP, below the detection limit of ICP-MS, were present in the myocardium. Given the high catalytic capacity of CeO_2_NP, these residual levels could contribute to the protective effects observed.

### Study limitations

The dose of DEPs used in this study was based in that employed by Soler-Segovia and coworkers in mice [29, 30], adjusted to our animals’ weight. The intratracheal dose administered in this study (7.5 mg/kg; corresponding to 1.875 mg for a 250 g rat) was converted to an equivalent 24-h airborne concentration. Assuming a typical minute ventilation of approximately 0.2 L/min for rats, the total inhaled air volume over 24 h (volume/minute * 60 min * 24 h) was estimated at 288 L or 0.288 m^3^. The equivalent air concentration **C** was calculated by applying the formula C = deposited dose/(inhaled volume*deposition efficiency), where the deposited dose was the complete amount of DEPs (1.875 mg), the inhaled volume was 0.288 m^3^, and the deposition efficiency was assumed to be 1 (or 100%). Accordingly, the equivalent air concentration was calculated to be approximately 6.51 mg/m^3^. This concentration was intentionally higher than levels expected from environmental PM2.5 levels (generally lower than 1 mg/m^3^ even under severe pollution episodes [76, 77]). This approach aligns with previous toxicological studies [78, 79], in which relatively high particle loads are administered to elicit measurable biological responses within short experimental time frames. Comparable dose ranges (2–10 mg/kg) have been employed in previous investigations of diesel exhaust particles and other ultrafine materials to elucidate mechanisms of inflammation and oxidative stress [40, 80, 81]. Nonetheless, it should be acknowledged that the dose used in the present study do not reflect typical ambient PM2.5 exposure levels, which may constrain the direct extrapolation of our findings to real-world scenarios. Although these results provide an initial proof-of-principle, they should be interpreted with caution, and further studies under more environmentally relevant exposure conditions are warranted.

Whole-body or nose-only inhalation represents the gold standard for studying inhalation toxicology. However, intratracheal instillation has been widely used in previous studies to investigate the pulmonary and systemic effects of particulate pollutants under controlled experimental conditions [79, 82]. In our study, both control and DEP-exposed animals underwent intratracheal administration, with the control group receiving saline, so any acute inflammatory response associated with the instillation procedure itself would be expected to occur equally in both groups. Therefore, the differences observed can be assumed to be specifically due to the particulate exposure rather than to the instillation process per se. Further, we acknowledge that this technique does not fully reproduce the chronic, low-grade exposure typical of real-world air pollution. However, it allows for precise dose control and targeted delivery to the lungs, which is particularly valuable for mechanistic studies like ours.

The pattern of changes in inflammatory and fibrotic markers observed in our study (elevation at 1 week followed by near-normal levels at 3 weeks) may suggest an acute phase response with subsequent resolution and repair. This observation supports the notion that repeated acute or subacute inflammatory events, rather than persistent low-grade inflammation, may also contribute to the development of cardiac remodeling and arrhythmogenesis in the context of DEP exposure.

The isolated heart model allows for precise investigation of the arrhythmogenic substrate within the cardiac muscle, offering an ethical advantage by reducing the number of animals required, as hearts can be studied even after appearance of persistent ventricular tachyarrhythmias, which would, otherwise, result in animal loses when arrhythmias are investigated in vivo. We acknowledge, however, that this model does not allow the investigation of the influence of the autonomic nervous system or spontaneous arrhythmic triggers, which represent an important limitation. Previous studies have already demonstrated that the autonomic nervous system plays a key role in the response to pollutant exposure, as demonstrated by altered heart rate variability [8, 83, 84]. Nevertheless, our findings provide complementary mechanistic insights that can be integrated with in vivo studies focused on arrhythmic triggers.

## Conclusions

In conclusion, prolonged exposure to DEPs has significant cardiac consequences, including pro-arrhythmic effects, myocardial inflammation, oxidative stress, and fibrosis, effects that were attenuated by CeO₂NPs, highlighting their potential as a therapeutic intervention. These findings highlight the crucial role of oxidative stress in DEP-related cardiac damage, and emphasize the impact of nanotherapeutics for preventing the cardiovascular complications associated with air pollution exposure.

## Supplementary Information

Additional file 1.

Additional file 2.

## Data Availability

Data underlying this article are available in bioRxiv at 10.1101/2025.04.11.648481v1, and will be available from the corresponding authors on reasonable request.

## Abbreviations

BA/BB: Benzyl alcohol/benzyl benzoate
BCL: Basic cycle length
CeO_2_NP: Cerium oxide nanoparticles
Cx43: Connexin 43
DEPs: Diesel exhaust particles
GSH/GSSG: Reduced to oxidized glutathione ratio
HW/TL: Heart weight to tibia length
MCP: Monocyte chemoattractant protein
MDA: Malondialdehyde
NAC: N-acetylcysteine
NSVT: Non-sustained ventricular tachyarrhythmias
PM: Particulate matter
PVB: Premature ventricular beats
ROS: Reactive oxygen species
SVT: Sustained ventricular tachyarrhythmias
TdP: Torsades de pointes
VT: Ventricular tachyarrhythmia
