# Supplementary material for: Insights into the causal role of diesel exhaust particles in ventricular arrhythmogenesis: protective effects of antioxidant cerium oxide nanoparticles
Source: Part Fibre Toxicol. 2025 Dec 25;22:36. doi: 10.1186/s12989-025-00649-2 (PMC12739854; doi:10.1186/s12989-025-00649-2)

INSIGHTS INTO THE CAUSAL ROLE OF DIESEL EXHAUST PARTICLES IN VENTRICULAR ARRHYTHMOGENESIS: PROTECTIVE EFFECTS OF ANTIOXIDANT CERIUM OXIDE NANOPARTICLES.

Western blot pictures

Figure 2C

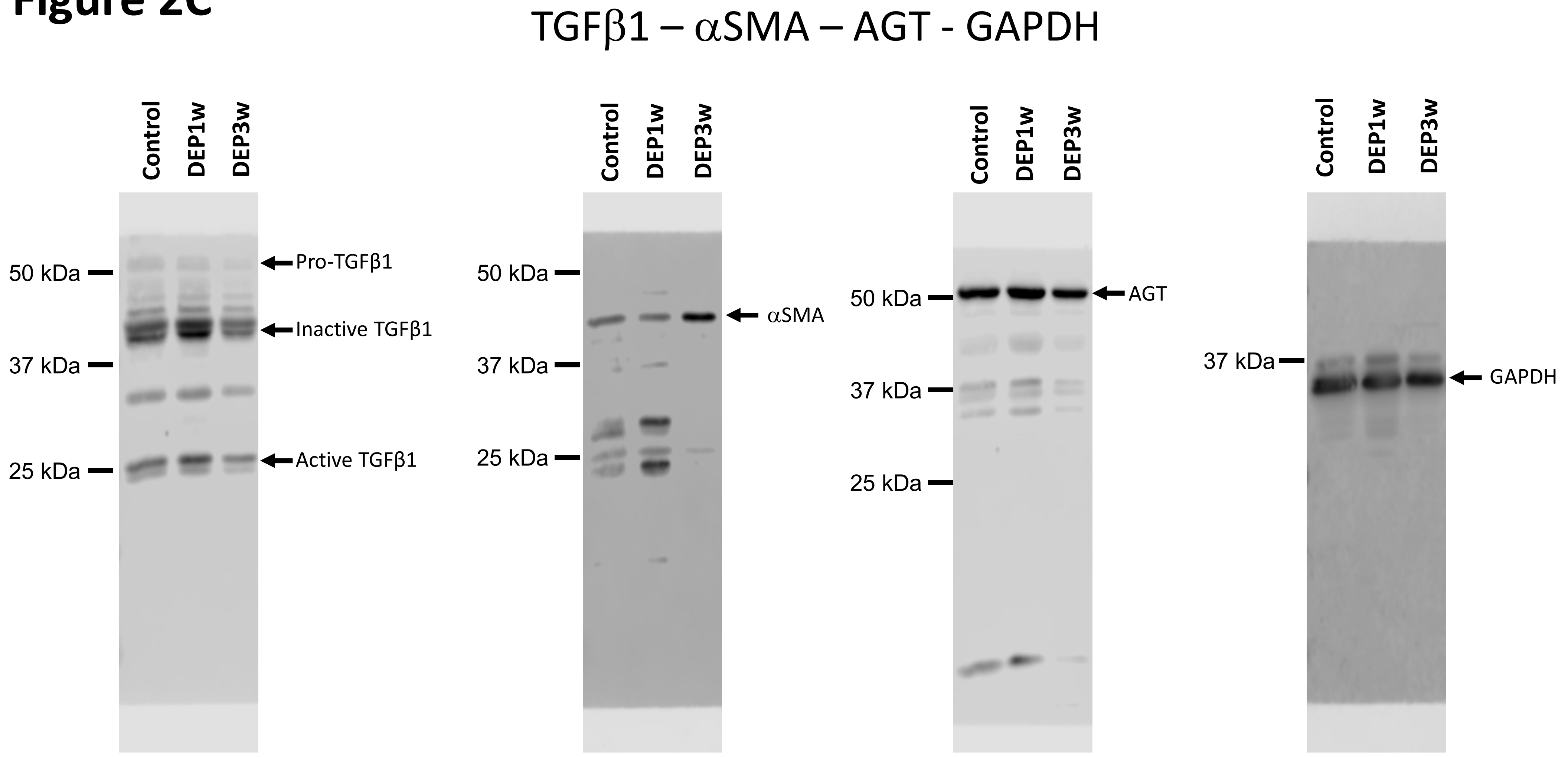

TGFβ1 – αSMA – AGT - GAPDH
Control
DEP1w
DEP3w
50 kDa
37 kDa
25 kDa
Pro-TGFβ1
Inactive TGFβ1
Active TGFβ1
αSMA
AGT
GAPDH

Figure 3B

NF-kB p65 -- GAPDH

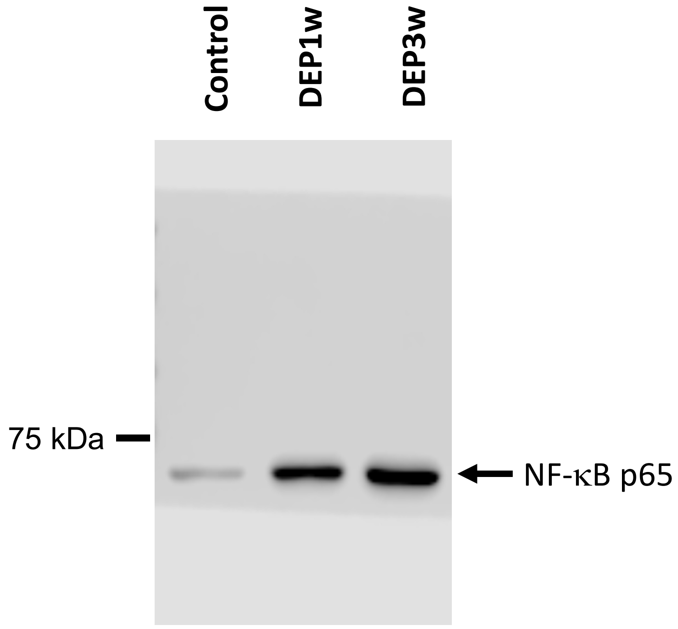

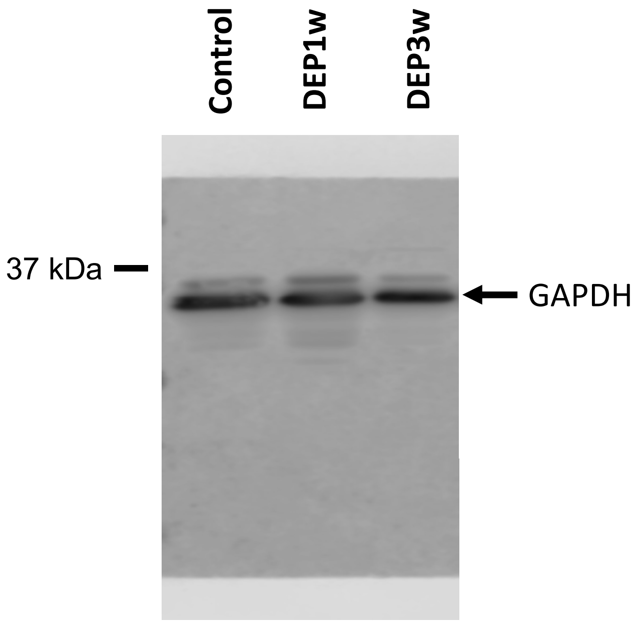

Supplemental Figure S5 A

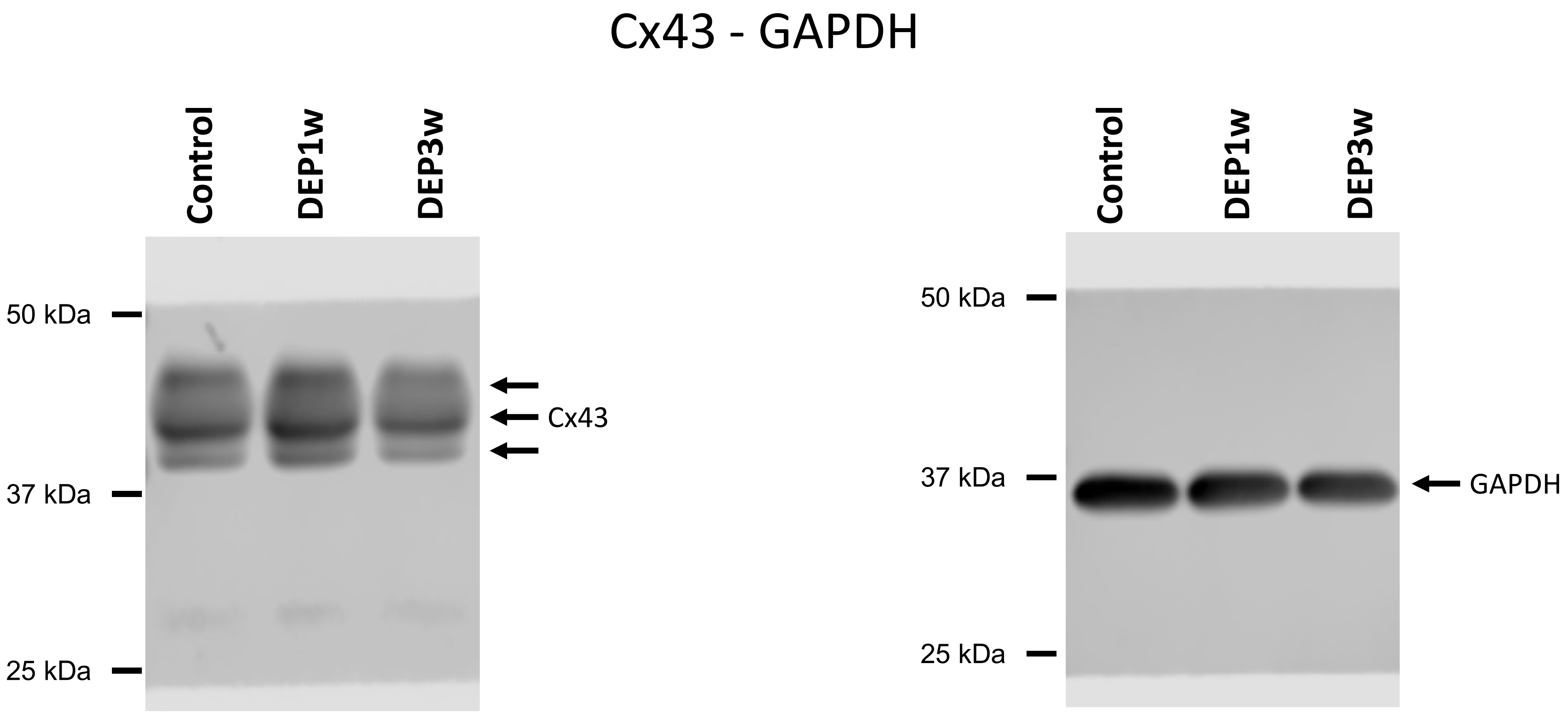

Supplemental Figure S6

AKT- ERK1/2-GAPDH

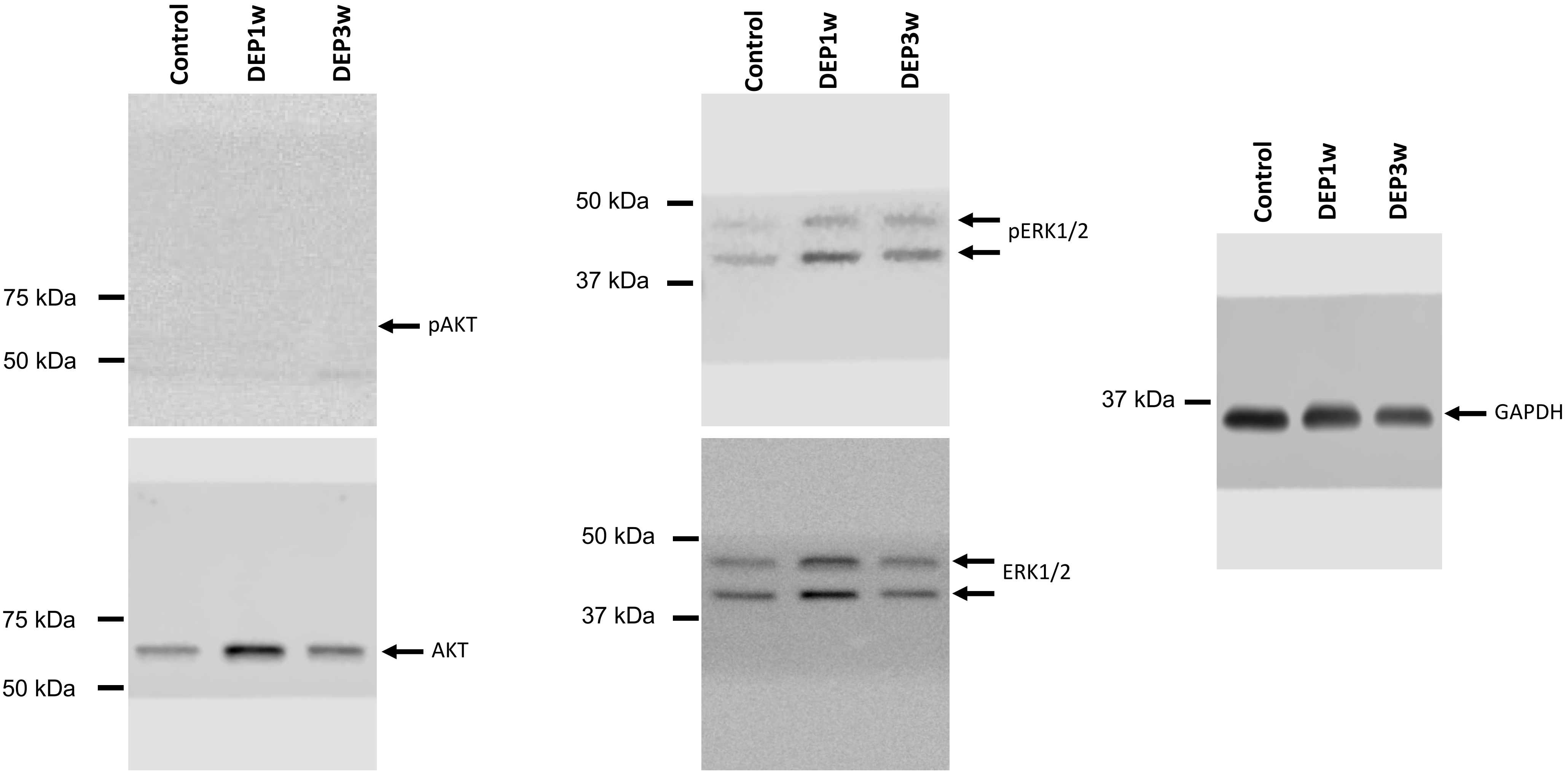

Supplemental Figure S6

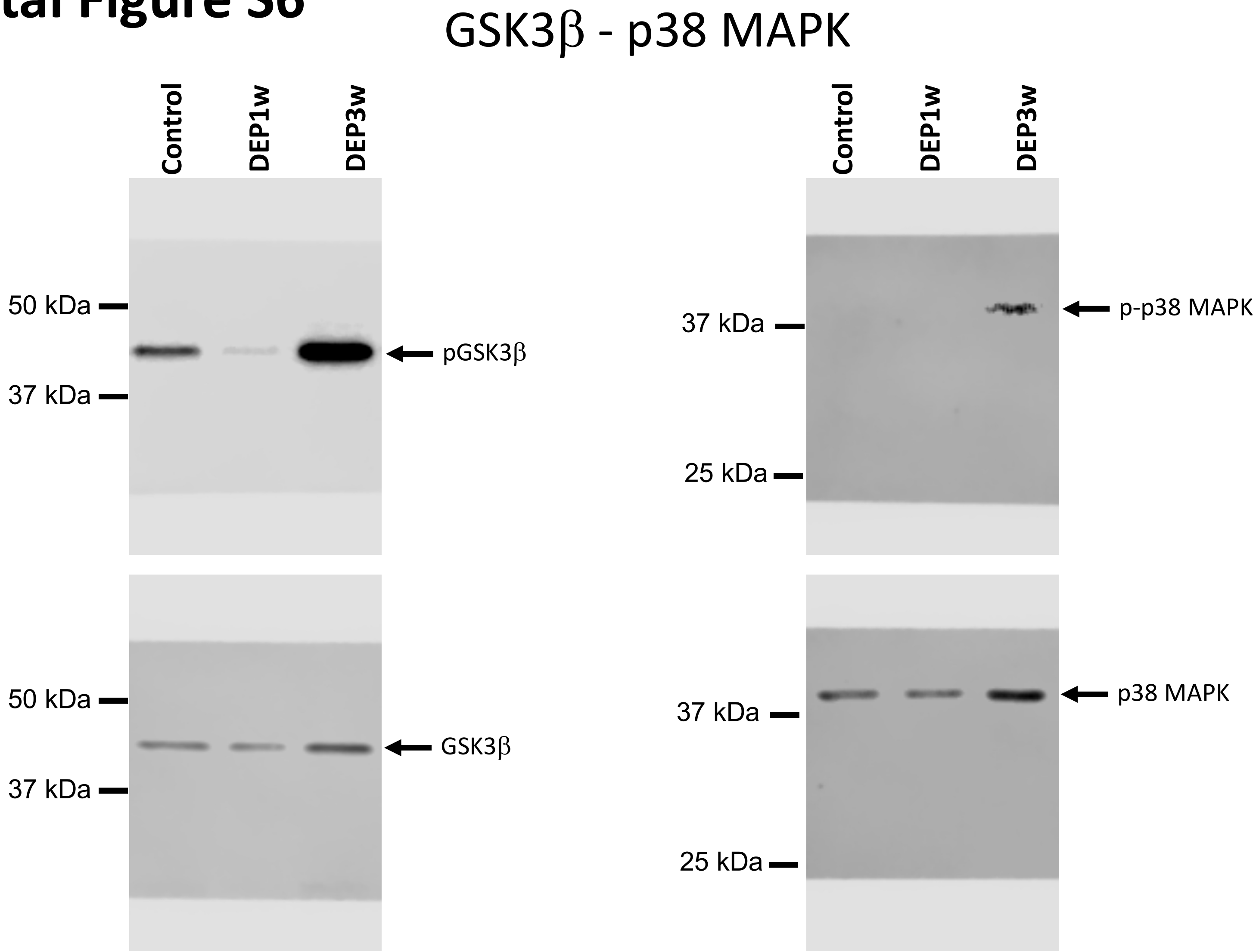

Supplemental Figure S7 A

SMAD2/3 - GAPDH

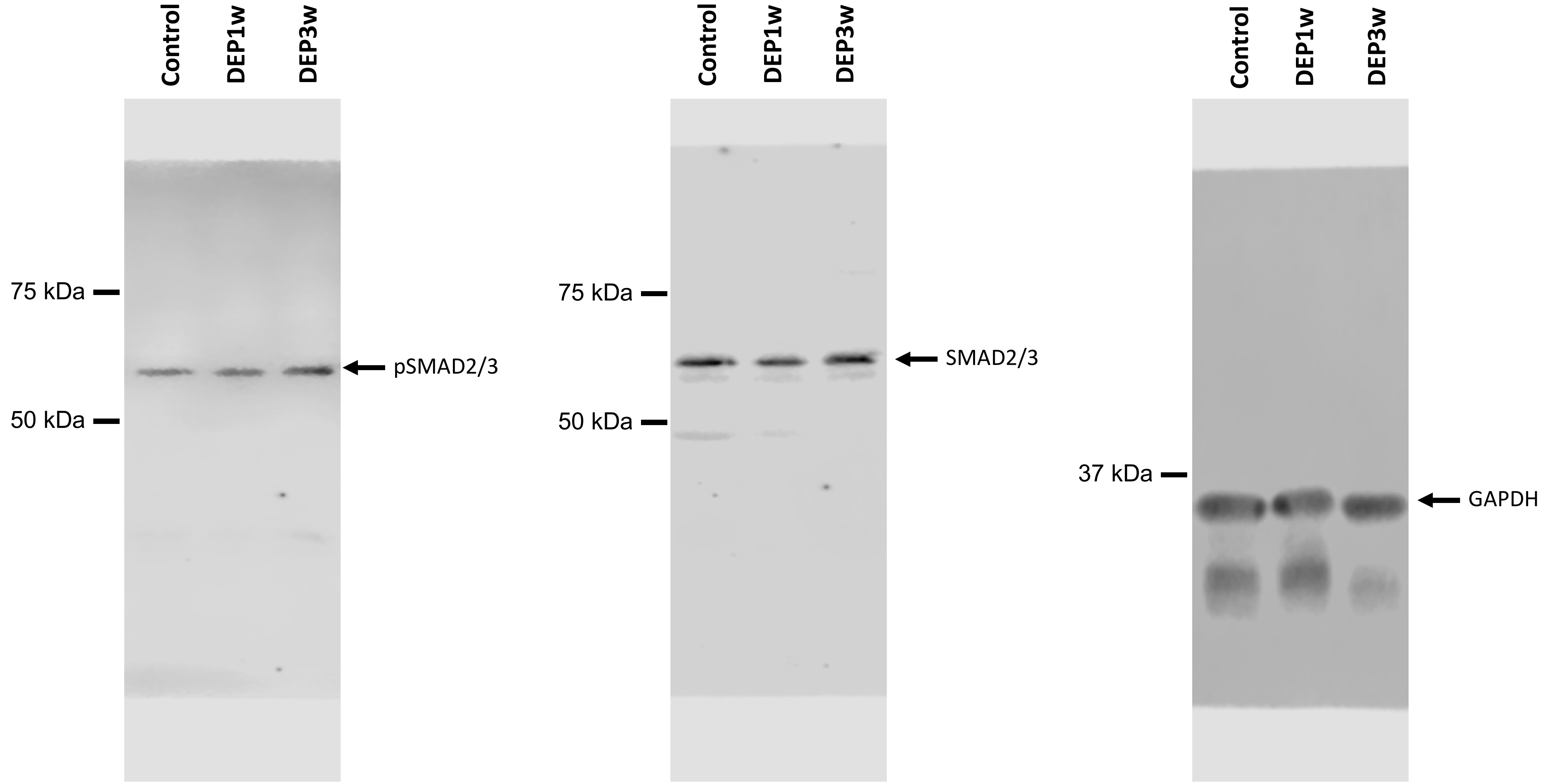

Supplemental Figure S7 B

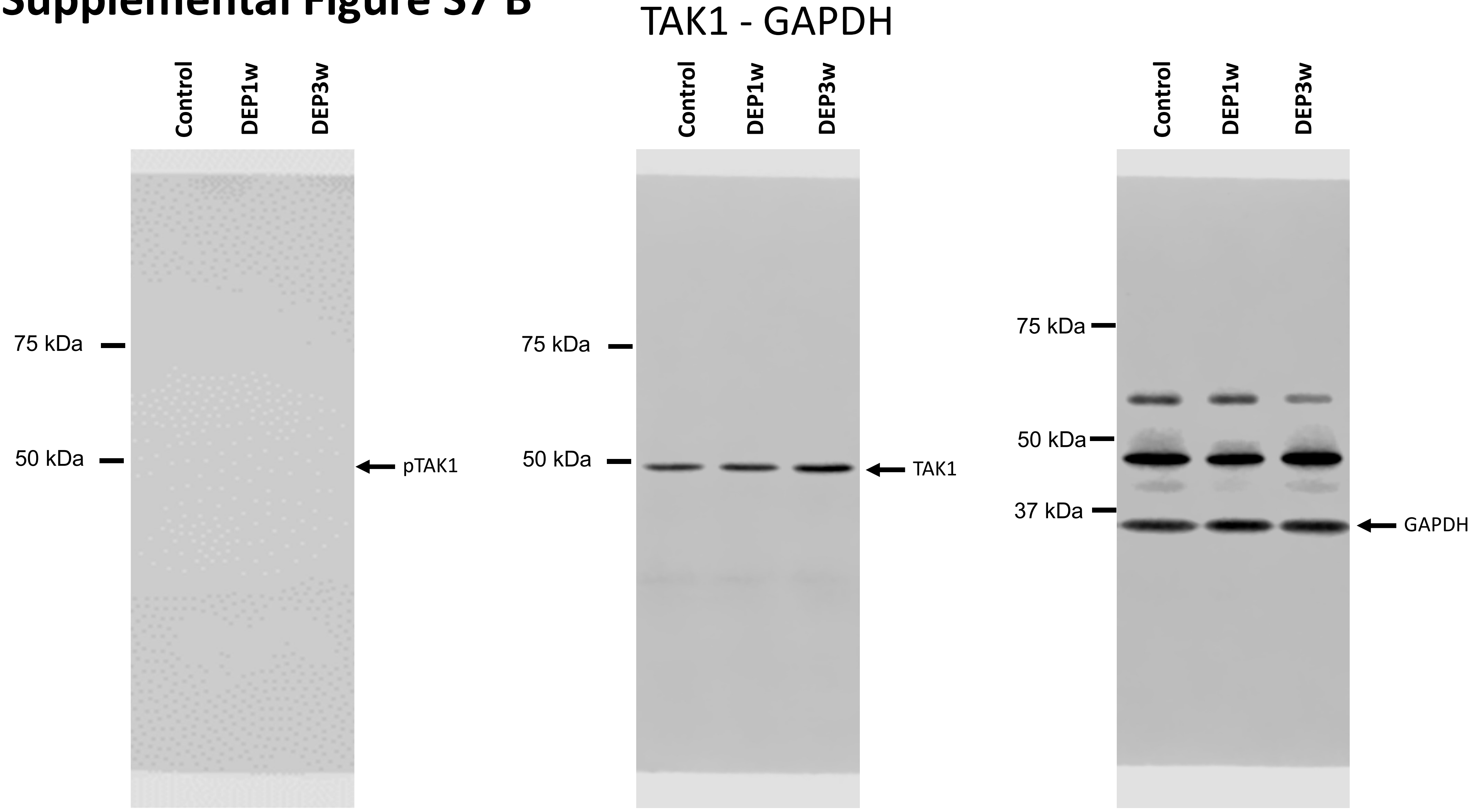

Supplement: Supplementary file 1 — Additional file 1. [file 12989_2025_649_MOESM1_ESM.pdf]
