# Supplementary material for: Insights into the causal role of diesel exhaust particles in ventricular arrhythmogenesis: protective effects of antioxidant cerium oxide nanoparticles
Source: Part Fibre Toxicol. 2025 Dec 25;22:36. doi: 10.1186/s12989-025-00649-2 (PMC12739854; doi:10.1186/s12989-025-00649-2)
Supplement: Supplementary file 2 — Additional file 2. [file 12989_2025_649_MOESM2_ESM.pdf]

SUPPLEMENTAL MATERIAL

INSIGHTS INTO THE CAUSAL ROLE OF DIESEL EXHAUST PARTICLES IN VENTRICULAR ARRHYTHMOGENESIS: PROTECTIVE EFFECTS OF ANTIOXIDANT CERIUM OXIDE NANOPARTICLES.

Freddy G. Ganse, ScD [1,2], Lena M. Ernst, ScD, PhD [3], Cristina Rodríguez, ScD, PhD [2,4], Marisol Ruiz-Meana, DVM, PhD [1,2], Javier Inserte, ScD, PhD [1,2], José Martínez-González, ScD, PhD [2,4,5], Ana M. Briones, ScD, PhD [2,6], Ana Belén García-Redondo, ScD, PhD [2,7], Marta Consegal, ScD, PhD [1,2], Elisabet Miró-Casas, ScD [1,2], Laia Yáñez-Bisbe, ScD, PhD [1,2], Aitor Pomposo, ScD [1,2], Marta Prades-Martínez, ScD [1,2], Ignacio Ferreira-González, MD, PhD [1,8], Victor Puntes, ScD, PhD [3,9,*], Begoña Benito, MD, PhD [1,2,*], Antonio Rodríguez-Sinovas, DVM, PhD [1,2,*].

[1] Cardiovascular Diseases Research Group, Vall d'Hebron Research Institute (VHIR), Barcelona, Spain.

[2] CIBER de Enfermedades Cardiovasculares (CIBERCV), Instituto de Salud Carlos III (ISCIII), Madrid, Spain.

[3] Disseny i Farmacodinàmica de Nanopartícules, Vall d'Hebron Research Institute, Barcelona, Spain; Catalan Institute of Nanoscience and Nanotechnology (ICN2), CSIC and BIST, Campus UAB, Bellaterra, 08193 Barcelona, Spain; Institució Catalana de Recerca I Estudis Avançats (ICREA), 08010 Barcelona, Spain.

[4] Institut de Recerca Sant Pau (IR SANT PAU), Barcelona, Spain.

[5] Instituto de Investigaciones Biomédicas de Barcelona-Consejo Superior de Investigaciones Científicas (IIBB-CSIC), Barcelona, Spain.

[6] Department of Pharmacology, Universidad Autónoma de Madrid, Instituto Investigación Hospital Universitario La Paz (IdiPaz), Madrid, Spain.

[7] Department of Physiology, Universidad Autónoma de Madrid, Instituto Investigación Hospital Universitario La Paz (IdiPaz), Madrid, Spain.

[8] CIBER de Epidemiología y Salud Pública (CIBERESP), Instituto de Salud Carlos III (ISCIII), Madrid, Spain.

[9] Networking Research Centre for Bioengineering, Biomaterials and Nanomedicine (CIBER-BBN), Instituto de Salud Carlos III (ISCIII), Madrid, Spain.

***Co-senior and Co-corresponding authors:**

Dr. Antonio Rodríguez-Sinovas (antonio.rodriguez.sinovas@vhir.org) and Dr. Begoña Benito (begona.benito@vhir.org), Cardiovascular Diseases Research Group, Vall d'Hebron University Hospital and Research Institute, Universitat Autònoma de Barcelona (Departament de Medicina), Pg. Vall d'Hebron 119, 08035 Barcelona, Spain. Phone: +34 937372442.

Dr. Víctor Puntes (victor.puntes@vhir.org), Disseny i Farmacodinàmica de Nanopartícules, Vall d'Hebron Research Institute, Barcelona, Spain; Catalan Institute of Nanoscience and Nanotechnology (ICN2), CSIC and BIST, Campus UAB, Bellaterra, 08193 Barcelona, Spain; Institució Catalana de Recerca I Estudis Avançats (ICREA), 08010 Barcelona, Spain.

SUPPLEMENTARY METHODS.

The data supporting the conclusions of this study are available from the corresponding authors. Animal studies complied with European legislation (Directive 2010/63/EU) on the protection of animals used for scientific purposes, with the Guide for the Care and Use of Laboratory Animals published by the US National Institutes of Health (NIH Publication No. 85-23, revised 1996, updated in 2011), and were approved by the Ethics Commettee of Vall d'Hebron Research Institute (protocol number CEEA49.20, CEA/11228/P1/1).

1. Preparation of diesel exhaust particles (DEPs).

DEPs (SRM-2975; National Institute of Standards and Technology, Gaithersburg, USA) were suspended in 0.9% sterile saline at a stock concentration of 20 mg/mL, vortexed and sonicated to minimize particle aggregation.

2. Preparation of CeO_2NP.

2.1. Synthesis of CeO_2NP.

Cerium (III) chloride heptahydrate (10 mM) was precipitated with tetramethylammonium hydroxide (27 mM) in the presence of sodium citrate (20 mM). The mixture was stirred overnight at room temperature and then refluxed at 100°C for 4 hours, yielding 3 nm CeO2 NPs (1.72 mg/mL). CeO2NPs were purified using 3 kDa centrifugal filters (Amicon-Ultra-15 3K, Merck) and resuspended in 2.2 mM sodium citrate.

2.2. CeO_2NP-RSA Conjugation.

To enhance biocompatibility, CeO_2NPs were conjugated with rat serum albumin (RSA, Merck) in 10 mM phosphate buffer (pH 7.4) at 4°C for 24 hours prior to injection.

2.3. Bacterial Endotoxin (LAL) test.

All synthesis and purification steps were performed under sterile conditions with non-pyrogenic materials. Quantitative determination of lipopolysaccharide (LPS) levels was conducted by Echevarne Analysis Laboratory (Barcelona, Spain).

2.4. Characterization of CeO_2NPs.

2.4.1. High-Resolution Transmission Electron Microscopy (HR-TEM).

The morphology and size of CeO_2NP were characterized using a Tecnai F20 S/TEM operated in high-resolution mode. For sample preparation, 10 μL of the nanoparticle suspension was drop-cast onto a 200-mesh carbon-coated copper grid and air-dried at room temperature. To ensure complete drying, samples were allowed to dry for a minimum of 24 hours. Nanoparticle size and size distribution were determined using ImageJ software. A minimum of 2000 particles were measured to ensure statistical significance (Supplementary Fig. S1).

2.4.2. UV-visible Spectroscopy.

UV-visible spectra of the nanoparticles were recorded using a Cary 60 spectrophotometer (Agilent Technologies, USA) within a wavelength range of 250-800 nm. Measurements were performed in 1.5 mL plastic cuvettes.

2.4.3. Dynamic Light Scattering (DLS) and ζ-Potential.

Hydrodynamic size and ζ-potential were determined using a Malvern Zetasizer Nano ZS (Malvern Instruments, UK) equipped with a 532 nm laser and a 173° backscatter detector. Measurements were conducted in 1 cm path length cells at 25°C, with three independent replicates per sample.

2.5. Organ Distribution and Cerium Content Determination.

Tissue samples were digested using an Ethos™ Easy microwave digestion system (Milestone). Samples were thawed and then combined with a 1:2 (v/v) nitric acid:water digestion solution. Digestion was performed at 200°C for 90 minutes. Cerium content was quantified by ICP-MS (Agilent 7900 ICP-MS) at the Chemical Analysis Service, UAB, Barcelona.

3. Experimental design and in vivo exposure to diesel exhaust particles and CeO_2NP.

Seventy-five Sprague-Dawley rats (both sexes, 1:1 ratio), aged 6 weeks at the start of the study, were used throughout the experiment. Following a four-week acclimatization period, the rats underwent intratracheal instillation of saline containing or not DEPs (7.5 mg/Kg, 0.375 mL/Kg). Instillations were administered under general anesthesia (ketamine (75 mg/Kg, IP) and medetomidine (0.5 mg/Kg, IP)) for one or three weeks, to

assess the effects of subacute and chronic exposure, respectively. Recovery from anesthesia was facilitated by atipamezole (1 mg/Kg, IP). The DEP dose and treatment regimen were adapted from Soler-Segovia et al. [1,2], scaled to the body weight of our animals. The influence of CeO_2 nanoparticles on the effects of DEP exposure were analyzed in additional rats weekly injected with CeO2NP (intraperitoneal; 0.5 mg/Kg the first week, 0.25 mg/Kg the second and third weeks, and 0.5 mg/Kg one day before sacrifice), beginning at the time of the first DEP exposure (Supplementary Fig. S2A). The dose of CeO_2 nanoparticles is in the same order of magnitude of that previously used for intravenous administrations in rats [3].

The pro-arrhythmic effects of DEPs were also assessed in rats with myocardial infarction (i.e., subjected to transient coronary occlusion within the 4 weeks prior to DEP exposure) (Supplementary Fig. S2B). Infarctions were induced as previously described [4], and the DEP exposure protocol was performed as described above. In brief, rats were anesthetized with ketamine (75 mg/kg, IP) and medetomidine (0.5 mg/kg, IP), intubated and mechanically ventilated (Inspira ASV, Harvard Apparatus). Animals were then placed in a Surgery Rodent Monitor (RSM+, Indus Instruments) that allows to maintain a core temperature of 37±0.5ºC while recording the ECG. Their hearts were exposed through a left thoracotomy at the fourth intercostal space, and the left anterior descending coronary artery (LAD) was transiently ligated using a 4/0 silk snare for a period of 60 minutes. The ligature was located 2 mm distal to the left atrial appendage. Successful performance of LAD occlusion was verified visually by the appearance of a pale color in the distal myocardium and by elevation of the ST segment at the ECG. Animals were then allowed to reperfuse until the time of DEP exposure (4 weeks after myocardial infarction). Following surgery, all animals received antibiotics (Enrofloxacine, 10 mg/Kg) and buprenorphine (0.05 mg/Kg every 8 h during the first 48 h), subcutaneously.

Successful DEP exposure was confirmed in the lungs of all experimental animals by fixation and dehydration in methanol, followed by incubation in a mixture of benzyl alcohol/benzyl benzoate (BA/BB) at a 2:1 ratio for at least one week. BA/BB rendered the lung tissue transparent, allowing visualization of the DEPs (Supplementary Fig. S2C). Additionally, part of the lungs was fixed in 4% formaldehyde, embedded in paraffin, and sectioned into 4 μm slices. Samples were stained with picrosirius red (Sigma-Aldrich,

MO, USA) and examined under a microscope (Eclipse Ts2R-FL, Nikon, Japan) at 40x magnification (Supplementary Fig. S2C).

4. Cardiac function by echocardiography.

Systolic cardiac function was assessed by transthoracic echocardiography using a Vivid Q portable ultrasound system equipped with a 13 MHz i12L-RS probe (GE Healthcare), as previously described [4]. Echocardiographic images were acquired at the end of the 3-week exposure period, one day before euthanasia. Rats were lightly anesthetized with 1.5% isoflurane, and images were captured in both long- and short-axis views. The following measurements were obtained in M-mode: left ventricular internal diameter in diastole (LVIDd) and systole (LVIDs), interventricular septum thickness in diastole (IVSd), left ventricular posterior wall thickness in diastole (LVPWd), fractional shortening (FS), and heart rate (HR). Ejection fraction (EF) was calculated using the formula: ((LVIDd - LVIDs) / LVIDd) * 100. Measurements were made by a researcher blinded to group allocation, and the final data presented for each parameter represent the average of 3–4 different cardiac cycles/animal.

5. Isolated, Langendorff-perfussed, rat heart preparation.

Three weeks after DEP or sham exposure, rats (13 weeks of age, males and females weighing 400–500 g and 250–300 g, respectively) were anesthetized with sodium pentobarbital (1.5 g/Kg, IP) and underwent a bilateral thoracotomy. Whole hearts were quickly excised and retrogradely perfused through the aorta with oxygenated (95% O2: 5% CO2) Krebs solution at 37°C (composition in mmol/L: NaCl 118, KCl 4.7, $MgSO_4$ 1.2, $CaCl_2$ 1.8, $NaHCO_3$ 25, KH_2PO_4 1.2, glucose 11, pH 7.4) in a constant flow Langendorff system, as previously described [5]. Perfusion flow (approximately 10 mL/min) was initially adjusted to produce a perfusion pressure of 50-60 mmHg under normoxic conditions. Left ventricular (LV) pressure was monitored using a water-filled latex balloon connected to a pressure transducer, placed in the left ventricle and inflated to maintain LV end-diastolic pressure (LVEDP) between 6 and 8 mmHg. All hearts were allowed to equilibrate for 30 minutes before the experiments.

6. Electrogram recordings.

Electrograms were recorded using stainless steel electrodes (Model 6491 unipolar pediatric temporary pacing lead, Medtronic France, Fourmies, France) placed at the left

ventricular base and at the metallic cannula used to perfuse the heart, as previously described [6]. A reference electrode was placed in a metallic part of the Langendorff system. Bipolar electrograms between the ventricular electrode and the cannula were used to monitor ventricular arrhythmias under normoxic conditions. Signals were amplified, digitized at 20 kHz, and stored for later analysis using a PowerLab/8SP Data Acquisition System and Chart 5.0 software (ADInstruments, Australia). Changes in the duration of the P wave, PR interval, QRS complex, and Hodges corrected QT interval were analyzed in these recordings.

7. Electrophysiological studies.

The effects of DEP exposure on the incidence of both spontaneous and inducible ventricular arrhythmias, together with the influence of CeO_2NP, analyzed, under normoxic conditions, in isolated hearts from rats previously exposed to saline or DEPs for three weeks. Spontaneous ventricular arrhythmias were studied during a 10-minute control period. Thereafter, inducible ventricular arrhythmias were triggered using two additional stainless steel electrodes placed at the cardiac apex and a protocol of programmed electrical stimulation, as previously described [6]. This protocol consisted of a train of 9 stimuli (S1), administered at a basic cycle length (BLC) of 150 ms, followed by 1 to 3 extrastimuli (S2-S4) (Fig. 1A). Pulse duration was set at 1 ms, and amplitude was set at a voltage double of the diastolic threshold. The extrastimuli interval was started at 60 ms, and was progressively shortened by 2 ms until the effective refractory period was reached. The effective refractory period was defined as the shortest interval that induced a propagated response at the electrode located at the ventricular base. The next extrastimulus was then applied at an interval 5 ms longer than the effective refractory period. The number and duration of premature ventricular beats (PVB), non-sustained ventricular tachyarrhythmias (NSVT, defined as those lasting less than 30 seconds), and sustained ventricular tachyarrhythmias (SVT, lasting more than 30 seconds) were recorded during the entire protocol. Ventricular tachyarrhythmias lasting more than 2 minutes were terminated by a brief (less than 2 minutes) period of global ischemia, in which case their duration was set at 120 seconds. The number of arrhythmic episodes was expressed as the count of events divided by the total stimulation cycles applied to each heart.

8. Heart weight to tibia length and interstitial collagen deposition.

Cardiac hypertrophy was calculated as the ratio of heart weight to tibia length (HW/TL) in those hearts used for arrhythmia induction. Interstitial collagen deposition was determined in additional animals treated for one or three weeks with saline containing or not DEPs. Cardiac slices from these animals were fixed in 4% formaldehyde, embedded in paraffin, and sectioned into 4 μm slices. Interstitial cardiac fibrosis was assessed after picrosirius red staining. At least eight random fields per animal, obtained from the left ventricle, were captured at 200x magnification using an Eclipse Ts2R-FL inverted microscope (Nikon, Japan) with a Leica DFC 550 digital camera. Interstitial collagen was evaluated using Image-J software (NIH, USA). The degree of interstitial fibrosis was calculated as the ratio of collagen surface area to total myocardial surface area (collagen volume fraction) [7]. In animals that were submitted to transient coronary occlusion, scar size was determined by quantifying the area of fibrosis stained with picrosirius red, and expressed as a percentage of the total slice area, normalized by cardiac weight.

9. Immunofluorescence analysis of connexin (Cx43) distribution.

Cx43 remodeling, including changes in expression and/or distribution, have a major influence on the appearance of cardiac arrhythmias [8]. Accordingly, we assessed Cx43 distribution by confocal laser scan microscopy in cryosections of cardiac samples from control and DEP-treated animals [6]. Cryosections were incubated with a polyclonal rabbit anti-Cx43 antibody (#70-0700, Zymed, Germany, dilution 1:50). Intercalated disks were identified with a monoclonal anti-pan Cadherin antibody (C1821, Sigma-Aldrich, dilution 1:500). Nuclei were stained with Hoeschst 33342 (10 μg/mL). Cx43 staining was performed in triplicate. Additionally, Cx43 expression was analyzed by conventional Western blot as described below.

10. Inflammatory cell infiltration.

Immunohistochemistry was performed in snap-frozen OCT-embedded cardiac sections (4 μm) from the additional hearts. Sections were first incubated, overnight (4°C), with a rabbit antibody raised against CD45 (ab10558, dilution 1:500, Abcam). Subsequently, the samples were incubated again for 90 min at room temperature with a biotinylated anti-rabbit secondary antibody (Vector Laboratories, Peterborough, UK). The standard Vectastain avidin-biotin peroxidase complex (ABC; Vector Laboratories, Peterborough, UK) was then applied. Color development was performed using 3,3′-diaminobenzidine

(DAB), and the sections were counterstained with hematoxylin before dehydration, clearing, and mounting. Negative controls, in which the primary antibody was omitted, were included to assess non-specific binding. All staining procedures were performed in duplicate.

11. Analysis of myocardial oxidative stress.

Oxidative stress was evaluated in myocardial samples from these additional animals by measuring the ratio of reduced to oxidized glutathione (GSH/GSSG) and the concentration of malondialdehyde (MDA), as a marker of lipid peroxidation.

Total GSH concentrations and those of the oxidized fraction (GSSG) were determined spectrophotometrically at 412 nm in myocardial extracts using the GSH reductase enzymatic method [9]. Briefly, frozen tissue extracts (50 µl) were thawed and assessed in phosphate-EDTA buffer containing GSH reductase (6 U/mL), NADPH (0.16 mg/mL) and DTNB (1.5 mg/mL). For GSSG measurement, duplicate samples were pre-incubated with 3 mmol/L 1-methyl-2-vinylpyridinium triflate, a thiol-scavenging reagent which selectively removes reduced GSH without affecting enzyme activity. The reduced GSH concentration was calculated by subtracting GSSG from the total GSH content. Results were expressed as nmoles of GSH per milligram of protein. A decrease in the GSH/GSSG ratio was used as an indicator of enhanced oxidative stress.

Additionally, lipid peroxidation, an indirect indicator of oxidative stress, was assessed by homogenizing myocardial tissue in ice-cold RIPA buffer and measuring MDA levels using a TBARS (TCA Method) Assay Kit (700870, Cayman Chemical), according to manufacturer's instructions. MDA concentrations were colorimetrically quantified at 430 nm using a multimode reader.

12. Real time-RT-qPCR.

Total RNA from the same additional rat hearts was isolated using the TriPure Isolation Reagent (Roche Diagnostics, Indianapolis, IN) according to the manufacturer's instructions. RNA integrity was determined by electrophoresis in agarose gels and was quantified using a NanoDrop 1000 Spectrophotometer (ThermoFisher Scientific, Waltham, MA). DNase I-treated total RNA (1 µg) was reverse-transcribed into cDNA using the High-Capacity cDNA Archive Kit (Applied Biosystems, Foster City, CA) with

random hexamers. Quantification of mRNA levels was performed by real-time qPCR using an ABI PRISM 7900HT sequence detection system (Applied Biosystems, Foster City, CA) and specific primers and probes for rat provided by Applied Biosystems (Assay-on-Demand system) as follows: atrial natriuretic peptide (NPPA; Rn00664637_g1), myosin heavy chain 7 (MYH7; Rn01488777_g1), collagen type I α1 chain (COL1A1; Rn01463848_m1), collagen type III α1 chain (COL3A1; Rn01437681_m1), lysyl oxidase (LOX; Rn00566984_m1), lysyl oxidase like 2 (LOXL2; Rn01466080_m1), transforming growth factor β (TGFβ1; Rn00572010_m1), matrix metallopeptidase 2 (MMP2; Rn01538170_m1), tumor necrosis factor α (TNFα; Rn99999017_m1), interleukin 1β (IL-1β; 00664637_g1), interleukin 6 (IL6; Rn01410330_m1), and EGF-like module-containing mucin-like hormone receptor-like 1 (EMR1; Rn01527631_m1). As endogenous control rat adenine phosphoribosyltransferase (APRT) was analyzed using specific primers (forward 5′-CGGGCGTGCTGTTCAGGGAT-3′ and Reverse 5′-TCAGGTGACCGGCCAGGAGG-3) and SYBR Green. Relative mRNA levels were determined using the $2^{-\Delta\Delta Ct}$ method.

13. Western blot analysis.

Protein expression was analyzed by conventional Western blot, as previously described [7]. Briefly, hearts were quickly frozen in liquid nitrogen, and homogenized in ice-cold buffer (NaCl 150 mmol/L, Trizma base 50 mmol/L, SDS 0.1%, sodium deoxycholate 0.5%, Triton X-100 1%, Tween20 0.1%, NaF 5 mmol/L, Na3VO4 1 mmol/L, and a protease cocktail inhibitor). The homogenate was centrifuged at 750 g for 10 minutes (4°C) to obtain a total tissue extract from the supernatant. Extracts were electrophoretically separated on 10-12% polyacrylamide gels. Proteins were then transferred to nitrocellulose membranes that were incubated with the primary antibodies of interest and the corresponding secondary antibodies. Immunoreactive bands were detected with ECL Prime Western Blotting Detection Reagent (Amersham Biosciences) and images were captured using an Odyssey FC Imaging System (LI-COR). Band intensities were measured by densitometry using the Image Studio Lite software.

The following antibodies were used: anti-Cx43 (Sigma-Aldrich, reference #C6219, dilution 1:8000), anti-ERK1/2 (Cell Signaling, #4695, 1:1000), anti-phospho-ERK1/2 (Cell Signaling, #9101, 1:500), anti-Akt (Cell Signaling, #9272, 1:1000), anti-phospho-

Akt (Cell Signaling, #9271, 1:500), anti-p38 MAPK (Cell Signaling, #8690, 1:1000), anti-phospho-p38 MAPK (Cell Signaling, #4511, 1:1000), anti-GSK3β (Cell Signaling, #9315, 1:1000), anti-phospho-GSK3β (Cell Signaling, #9323, 1:500), anti-SMAD2/3 (Cell Signaling, #5678S, 1:1000), anti-phospho-SMAD2/3 (Abcam, #ab272332, 1:1000), anti-TAK1 (Cell Signaling, #5206S, 1:1000), anti-phospho-TAK1 (Cell Signaling, #4508S, 1:1000), anti-TGFβ1 (Abcam, #ab92486, 1:1000), anti-αSMA (Sigma-Aldrich, #A5228, 1:1000), anti-NF-κB p65 (Abcam, #ab16502, 1:1000), anti-angiotensin receptor (AGT) (Biorbyt, #orb422803, 1:1000). Glyceraldehyde-3-phosphate dehydrogenase (GAPDH) (GeneTex, #GTX627408, 1:5000) was used as loading control.

14. Statistics.

Data are expressed as mean±SEM. Differences in the number of spontaneous or induced ventricular arrhythmias were assessed using nonparametric Mann-Whitney or Kruskal-Wallis and Dunn's tests. Additional analyses were performed by two-way ANOVA to determine the effects of previous myocardial infarction and DEP exposure or the impact of sex. Incidences were assessed by χ^2 or Fisher's exact tests. Changes in the heart weight-to-tibia length ratio (HW/TL), echocardiographic measurements, collagen deposition, inflammatory cell infiltration, RT-qPCR, and Western blot data were analyzed using Student's t-test or one-way ANOVA with Tukey post-hoc tests. Differences were considered significant when $p<0.05$.

References.

1. Soler-Segovia D, de Homdedeu M, Sánchez-Díez S, Romero-Mesones C, Espejo D, Marain F, et al. Immunological Effects of Diesel Particles in a Murine Model of Healthy Mice. Toxics. 2024;12:530.

2. Alvarez-Simon D, Munoz X, Gomez-Olles S, de Homdedeu M, Untoria MD, Cruz MJ. Effects of diesel exhaust particle exposure on a murine model of asthma due to soybean. PLoSOne. 2017;12:e0179569.

3. Córdoba-Jover B, Arce-Cerezo A, Ribera J, Pauta M, Oró D, Casals G, et al. Cerium oxide nanoparticles improve liver regeneration after acetaminophen-induced liver injury and partial hepatectomy in rats. J Nanobiotechnology. 2019;17:112.

4. Poncelas M, Inserte J, Aluja D, Hernando V, Vilardosa U, Garcia-Dorado D. Delayed, oral pharmacological inhibition of calpains attenuates adverse post-infarction remodelling. Cardiovasc Res. 2017;113:950–61.

5. Valls-Lacalle L, Barba I, Miro-Casas E, Alburquerque-Bejar JJ, Ruiz-Meana M, Fuertes-Agudo M, et al. Succinate dehydrogenase inhibition with malonate during reperfusion reduces infarct size by preventing mitochondrial permeability transition. Cardiovasc Res. 2016;109:374–84.

6. Sanchez JA, Rodriguez-Sinovas A, Fernandez-Sanz C, Ruiz-Meana M, Garcia-Dorado D. Effects of a reduction in the number of gap junction channels or in their conductance on ischemia-reperfusion arrhythmias in isolated mouse hearts. Am J Physiol Heart Circ Physiol. 2011;301:H2442–53.

7. Valls-Lacalle L, Negre-Pujol C, Rodriguez C, Varona S, Valera-Canellas A, Consegal M, et al. Opposite Effects of Moderate and Extreme Cx43 Deficiency in Conditional Cx43-Deficient Mice on Angiotensin II-Induced Cardiac Fibrosis. Cells. 2019;8:1299.

8. Rodríguez-Sinovas A, Sánchez JA, Valls-Lacalle L, Consegal M, Ferreira-González I. Connexins in the Heart: Regulation, Function and Involvement in Cardiac Disease. Int J Mol Sci. 2021;22:4413.

9. Owen JB, Butterfield DA. Measurement of oxidized/reduced glutathione ratio. Methods Mol Biol. 2010;648:269–77.

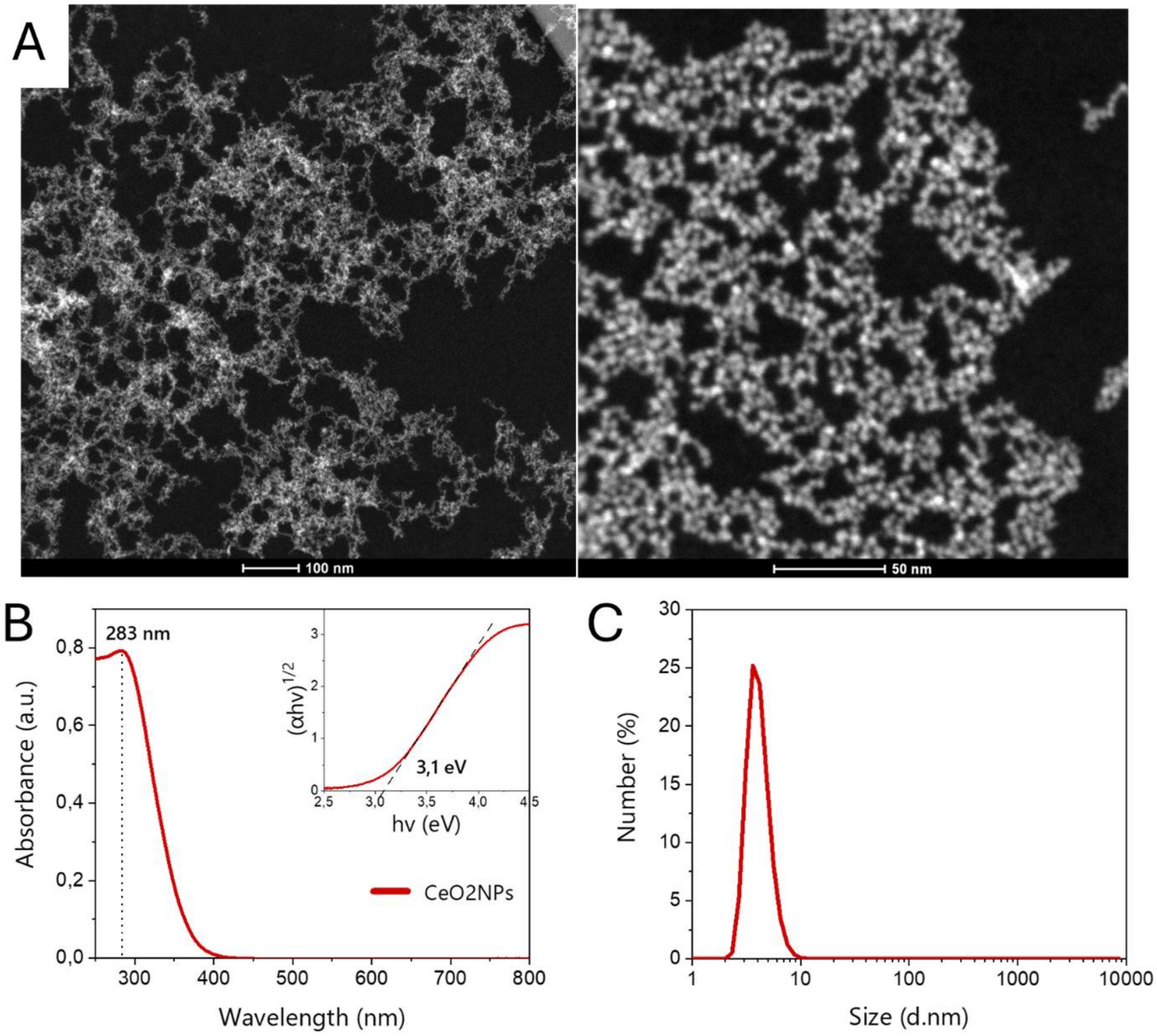

Supplemental Figure S1. Characterisation of CeO_2NPs. **A)** Representative High Annular Dark Field (HAADF) and High-Resolution (HRES) Transmission Electron Microscopy (TEM) images. Scale bar = 100 and 50 nm, respectively. **B)** UV-Vis spectra of CeO_2 NPs and their representation as Tauc plot from the UV–Vis analysis in the inset were the intersection of the two fitting lines estimates the band gap energy. **C)** DLS spectra of CeO_2NP by number distribution.

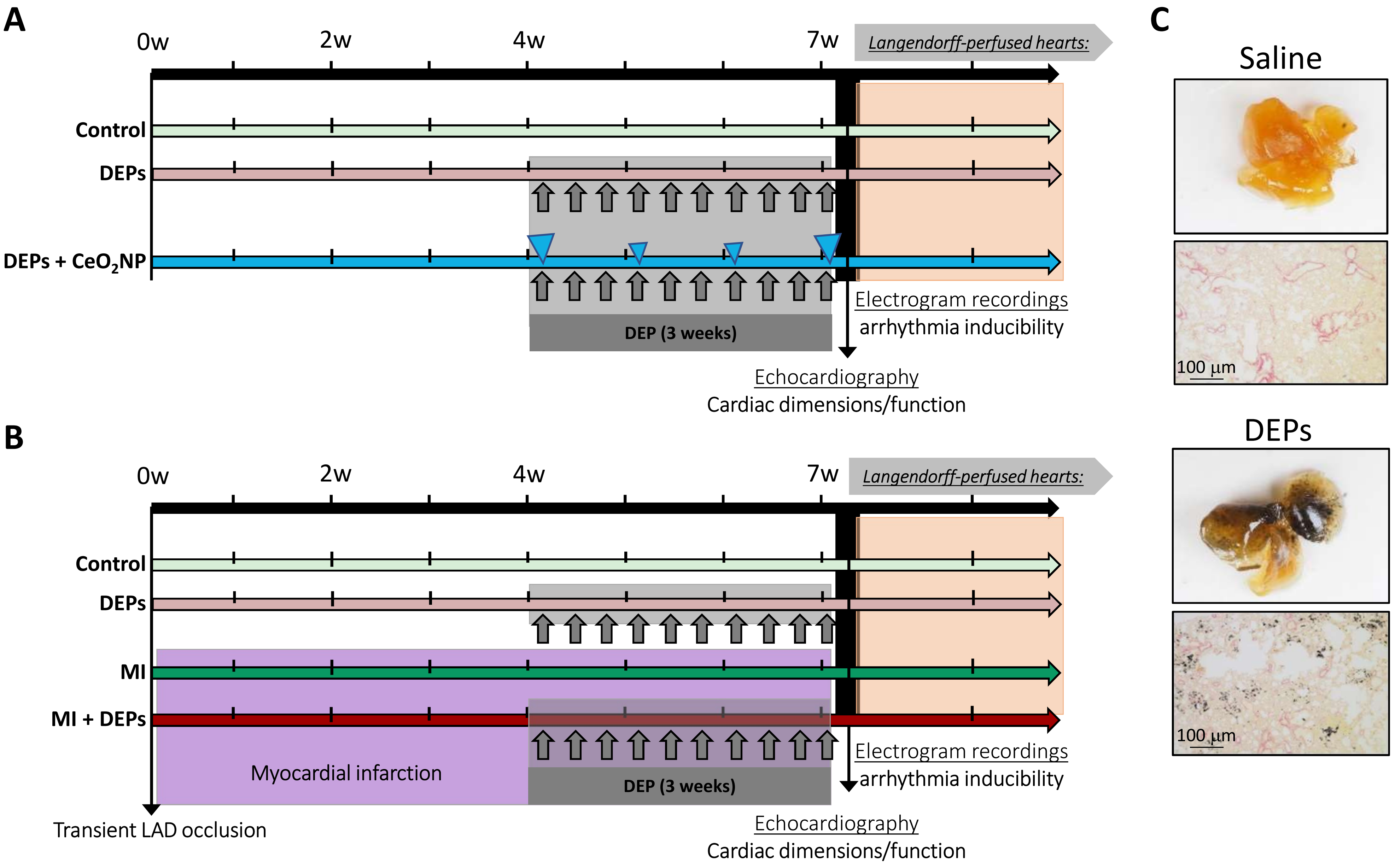

Supplemental Figure S2: Outline of the experimental protocols used in the study (A, B). (C) Shows macroscopic and microscopic images of lungs from a saline-treated animal and from a rat intratracheally instilled with saline for three weeks. Macroscopic images were obtained after incubation with benzyl alcohol/benzyl benzoate (BA/BB) for transparentation. Microscopic images were stained with Picrosirius Red. Grey arrows indicate the timing of DEP instillation (monday, wednesday and Friday, up to a total of 10 administrations). Big and small blue triangles indicate administation of CeO_2 nanoparticles (0.5 and 0,25 mg/Kg, respectively).

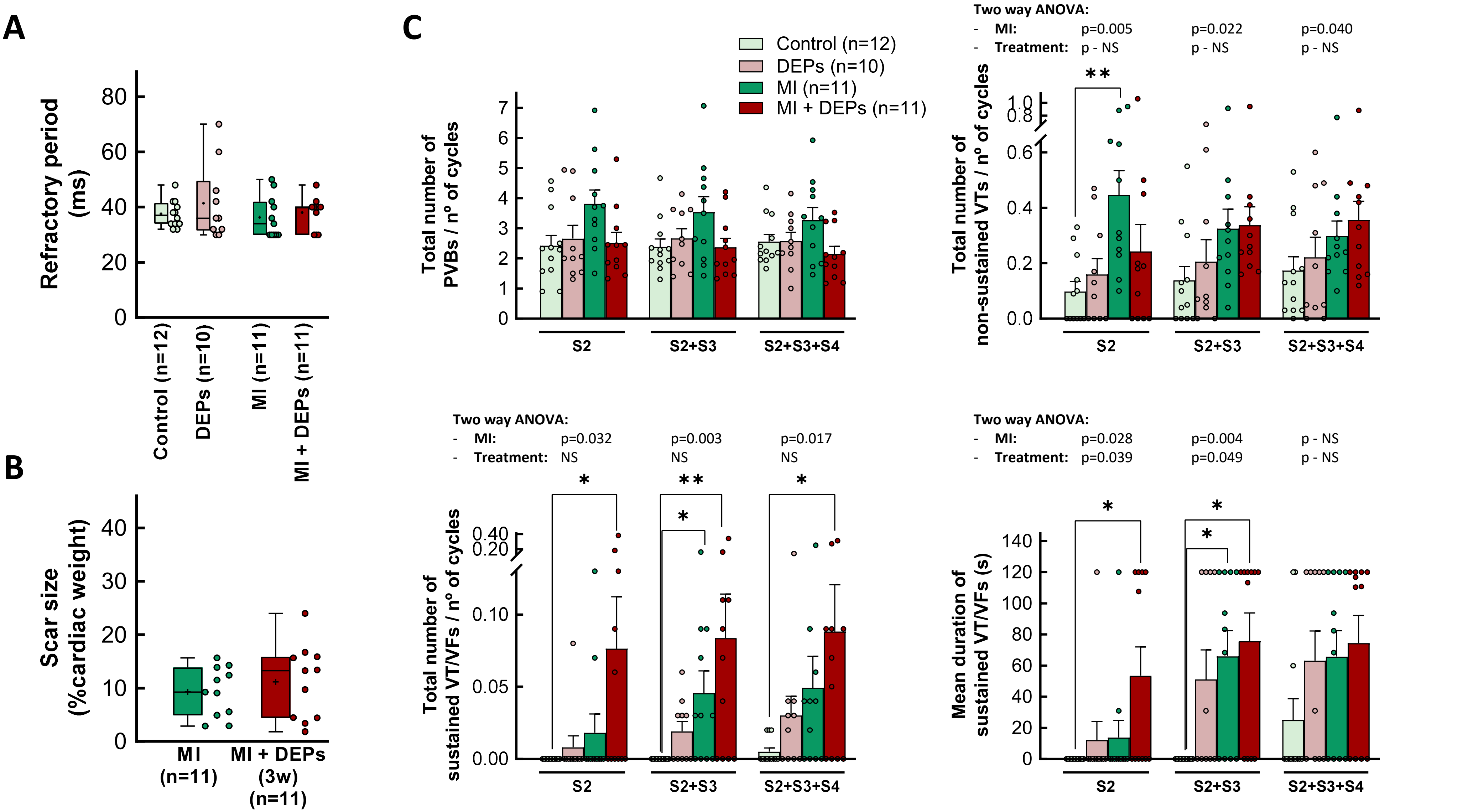

Supplemental Figure S3: Inducibility of ventricular arrhythmias in hearts from infarcted rats exposed to DEPs. (A) Refractory periods in isolated hearts from control and DEP-exposed animals either submitted or not to myocardial infarction (MI). (B) Scar size in animals with myocardial infarction and intratracheally instilled, four weeks later, with saline contaning or not DEPs. (C) Number of premature ventricular beats (PVBs), non-sustained tachycardias (VTs) and sustained tachyarrhythmias detected after application of 1, 2 or 3 extrastimuli, expressed relative to the number of stimulation cycles, in isolated rat hearts from animals intratracheally instilled for three weeks with saline or DEPs, either submitted or not to myocardial infarction. Mean duration of sustained tachyarrhythmias is shown at the right bottom. Significance tested by Kruskal-Wallis and Dunn's tests.* ($p<0.05$) and ** ($p<0.01$) indicate significant differences vs. indicated groups (Krustal-Wallis and Dunn's tests). Additionally, significance was tested by two-way ANOVA, as indicated in each figure.

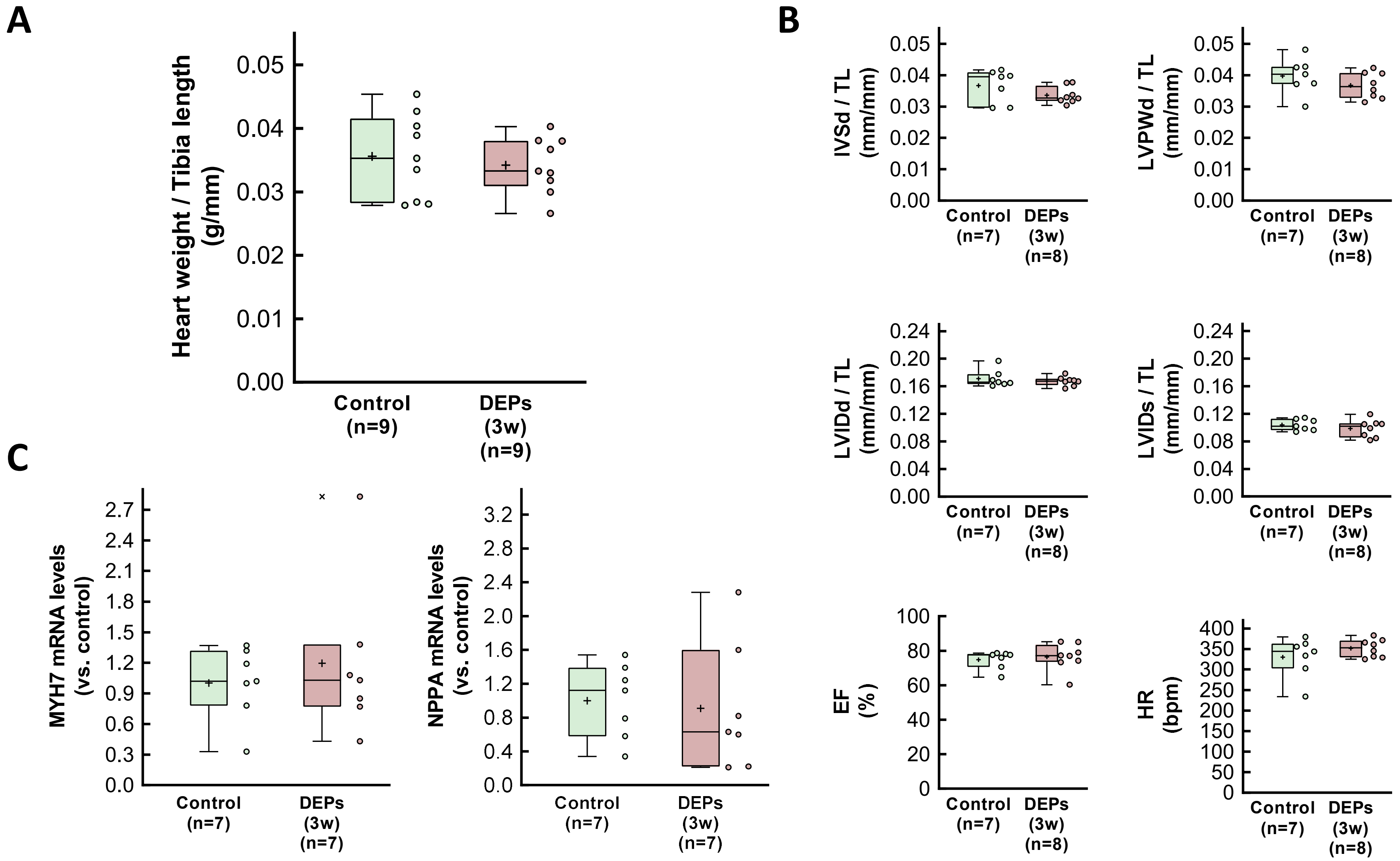

Supplemental Figure S4: Assessment of myocardial hypertrophy in DEP-exposed rats. (A) Heart weight expressed respect to tíbia length in hearts from control and DEP-exposed animals. (B) Changes in interventricular septum thickness during diastole (IVSd), left ventricular posterior wall thickness during diastole (LVPWd), left ventricular end-diastolic internal diameter (LVEDD), left ventricular end-systolic internal diameter (LVESD), ejection fraction (EF), and heart rate in control rats and in animals intratracheally instilled with DEPs for three weeks. IVSd, LVPWd, LVEDD and LVESD are expressed respect to tibia length. (C) Myocardial levels of mRNAs coding for proteins involved in the hypertrophic response (MYH7 and NPPA) analyzed in tissue extracts from the same experimental groups. Data are shown as box plot depicting median (horizontal line), mean (+), and individual values (color symbols). Outlayers are marked with “x”, and were also included in the analyses. Significance tested by Student’s t test.

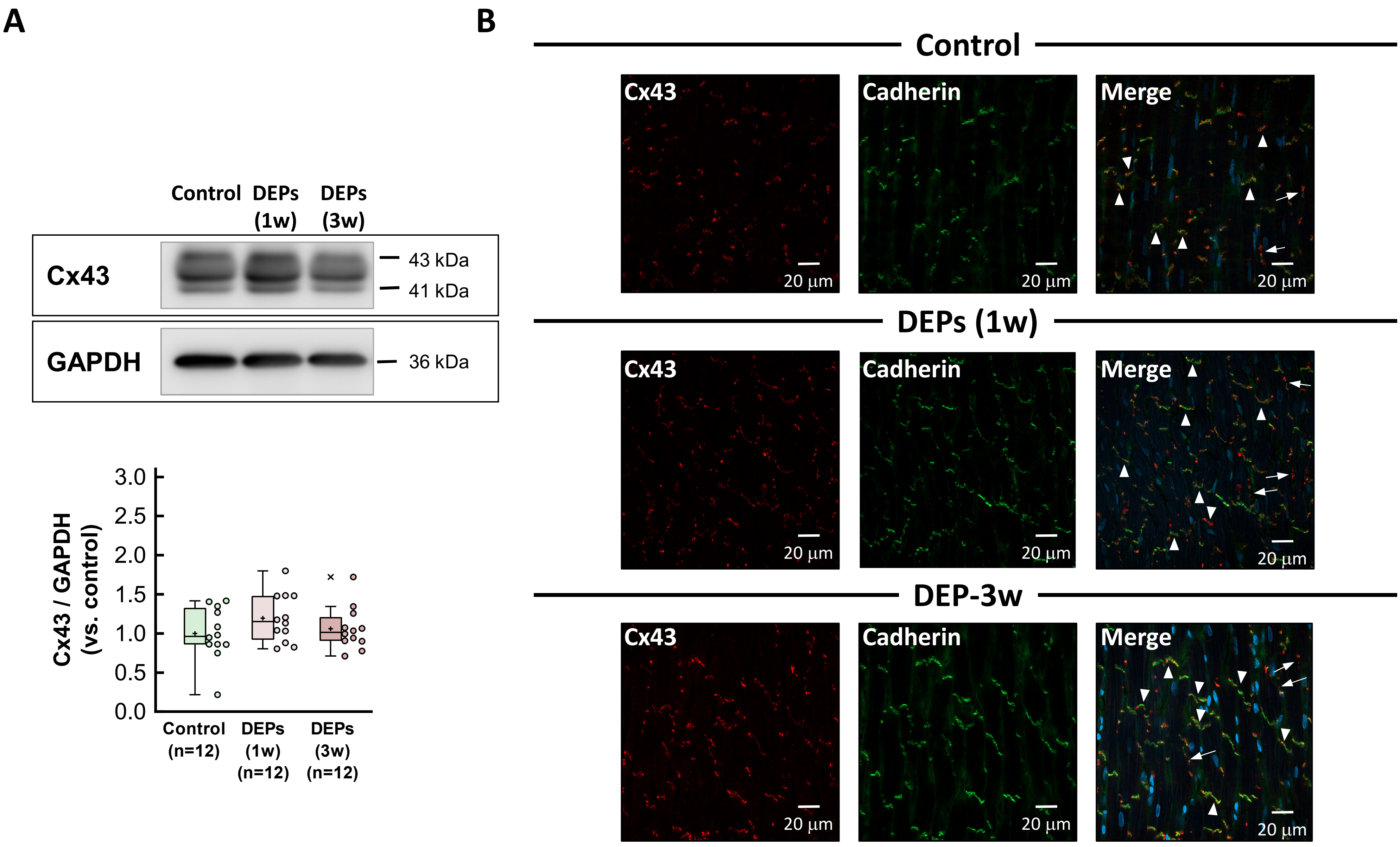

Supplemental Figure S5: Analysis of expression and distribution of Cx43. (A) Representative Western blot showing changes in Cx43 expression in myocardial samples from control animals and from rats exposed to DEPs for 1 or 3 weeks. Quantification is shown below. Data are shown as box plot depicting median (horizontal line), mean (+), and individual values (color symbols). Outlayers are marked with "x", and were also included in the analyses. Significance tested by one-way ANOVA followed by Tukey post-hoc test. (B) Confocal microscopy images showing distribution of Cx43 and pan-Cadherin in myocardial samples from the same experimental groups. Arrow heads indicate presence of Cx43 at the intercalated disc, where it co-locatizes with pan-Cadherin. Arrows indicate residual lateralization.

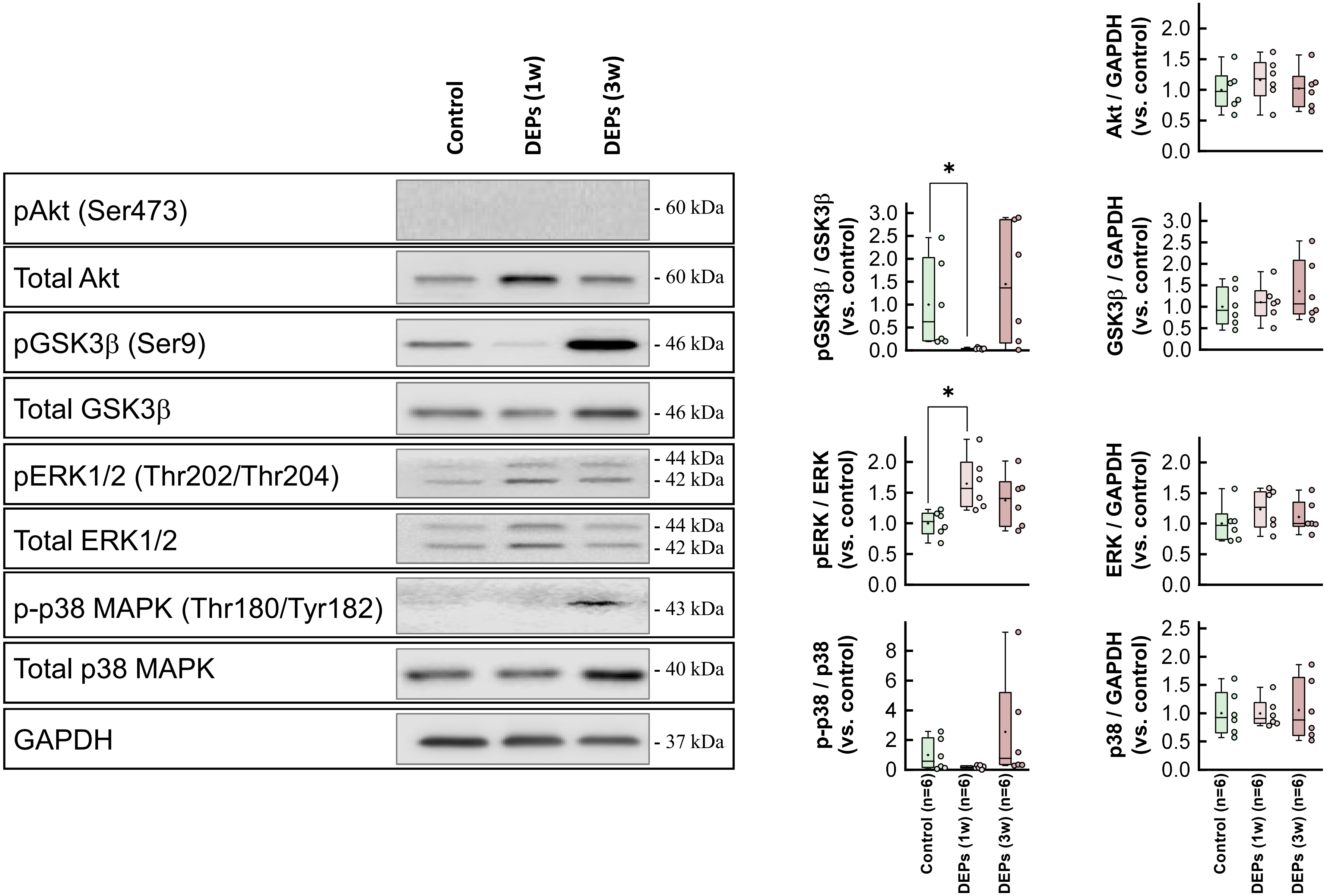

Supplemental Figure S6: Changes in the expression of proteins involved in cytosolic sygnaling cascades in hearts from DEP-exposed rats. Representative Western blot analysis showing expression and degree of activation (i.e., phosphorylation) of Akt, GSK3b, ERK1/2 and p38 MAPK in myocardial samples from control animals and from rats exposed to DEPs for 1 or 3 weeks. Quantifications are shown at the right. No activation was detected for Akt in any case. Data are shown as box plot depicting median (horizontal line), mean (+), and individual values (color symbols). Significance tested by one-way ANOVA followed by Tukey post-hoc test.* ($p<0.05$) show significant differences vs. indicated groups.

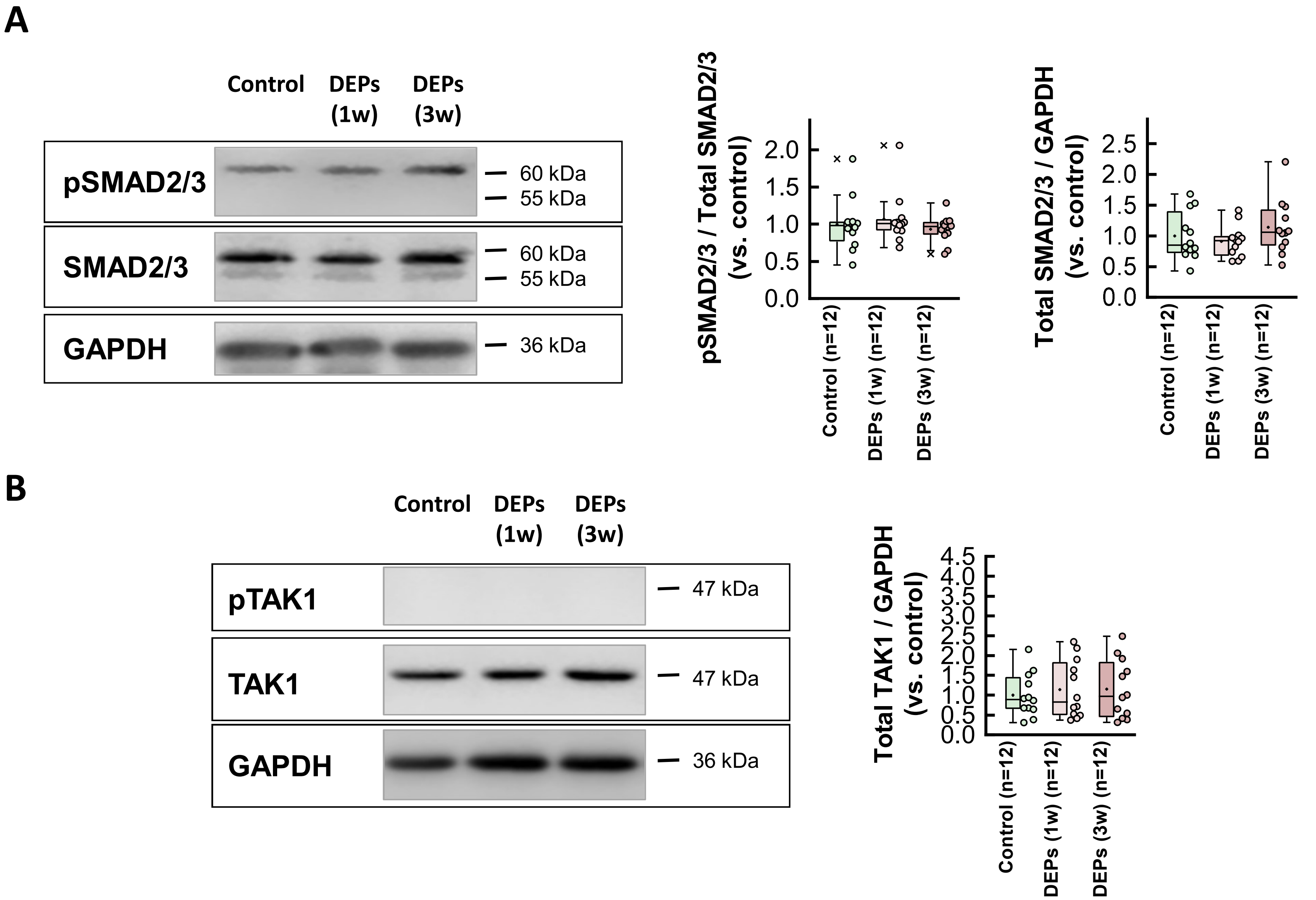

Supplemental Figure S7: Changes in the expression of SMAD2/3 and TAK1 in hearts from DEP-exposed rats. (A) Representative Western blot showing expression and degree of activation of SMAD2/3, protein potentially involved in the fibrotic response, in myocardial samples from control animals and from rats exposed to DEPs for 1 or 3 weeks. Quantifications appear at the right. Data are shown as box plot depicting median (horizontal line), mean (+), and individual values (color symbols). Outlayers are marked with "x", and were also included in the analyses. (B) Representative Western blot showing expression and degree of activation of TAK1. Only total expression was quantified as no phosphorylation was detected in these samples. Significance tested by one-way ANOVA followed by Tukey post-hoc test.

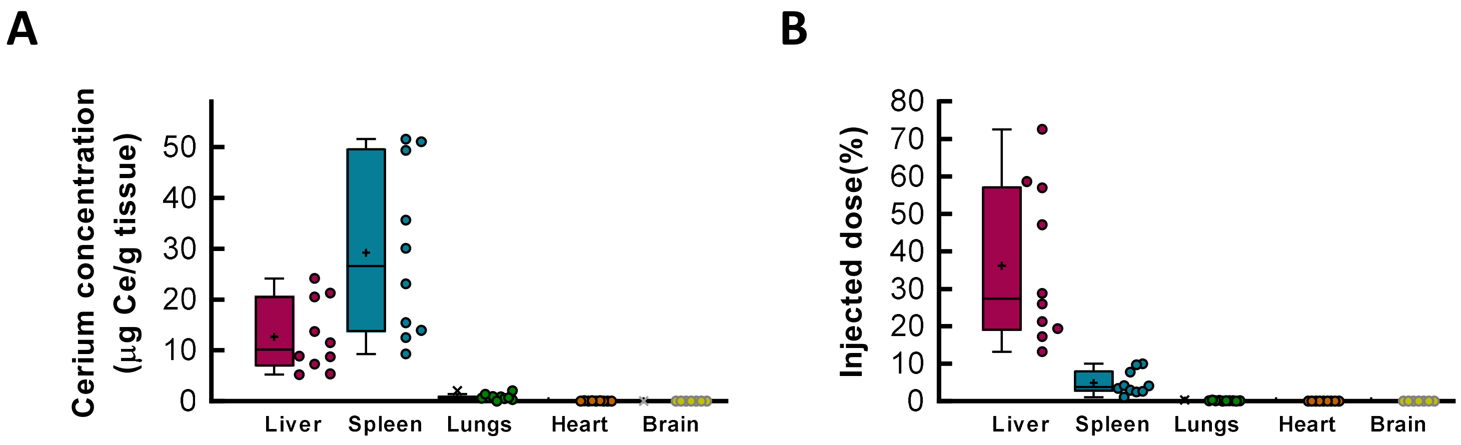

Supplemental Figure S8: Tissue cerium concentrations measured by ICP-MS 22 days after the first CeO_2NP intraperitoneal injection (2x 0.5 mg/Kg and 2x 0.25 mg/Kg). A. Cerium concentrations, in µg Ce/g tissue. B. Cerium concentrations expressed as a percentage of the injected dose. (liver, spleen, lungs, n = 10; Heart, n = 9; Brain, n = 7).
